# Why AGG is associated with high transgene output: passenger effects and their implications for transgene design

**DOI:** 10.1093/nargab/lqaf086

**Published:** 2025-06-19

**Authors:** Kate G Daniels, Sofia Radrizzani, Laurence D Hurst

**Affiliations:** Milner Centre for Evolution, Department of Life Sciences, University of Bath, Bath, BA2 7AY, UK; Milner Centre for Evolution, Department of Life Sciences, University of Bath, Bath, BA2 7AY, UK; Milner Centre for Evolution, Department of Life Sciences, University of Bath, Bath, BA2 7AY, UK

## Abstract

In bacteria, high A and low G content of the 5′ end of the coding sequence (CDS) promotes low RNA stability, facilitating ribosomal initiation and subsequently a high protein to transcript ratio. Additionally, 5′ NGG codons are suppressive owing to peptidyl-tRNA drop off. It was, therefore, surprising that the first large-scale transgene experiment to interrogate the 5′ effect by codon randomization found the NGG, G-rich codon AGG to be the most associated with high transgene output. Why is this? We show that this is not replicated in other large transgene datasets, where AGG and NGG are associated with low efficiency. More generally, there is limited agreement between the first experiment and others. This we find to be a consequence of non-random construct design. In constructs of the first experiment, AGG disproportionately occurs with non-AGG codons associated with low stability and high protein output, making AGG’s association with high output an artefact. While translationally non-optimal codons like AGG are conjectured to slow ribosomes for orderly initiation, we find that in the less biased constructs high, not low, translational adaptation in the first 10 codons is (weakly) predictive of higher translational efficiency. These results have implications for both transgene and experimental design.

## Introduction

“*Treasure your exceptions! When there are none, the work gets so dull that no one cares to carry it further. Keep them always uncovered and in sight. Exceptions are like the rough brickwork of a growing building which tells that there is more to come and shows where the next construction is to be*.” Bateson 1908 [1].

Understanding why transgenes that differ exclusively at synonymous sites differ by orders of magnitude in their resultant protein level (and proteins per transcript) is a central concern for comprehension of the causes of selection on synonymous mutations, hence codon usage, for determining the factors affecting the transcription and translation processes and for biotechnology, most notably transgene protein production [2–11].

In *E. coli*, transgene experiments routinely point to the disproportionate influence of the most 5′ codons in the coding sequence (CDS) on protein production [2–5, 7, 8, 11–14]. Highly expressed transgenes (tHEGs) tend to be A rich and G poor at 5′ synonymous sites, this producing a low stability mRNA 5′ end [3, 4, 11, 15, 16]. A can make only weak bonds with U, while G makes strong bonds with C and weak bonds additionally with U via non-canonical G:U pairing [15]. There are multiple mutually compatible reasons why low 5′ mRNA stability might promote translation: it means less work for the ribosome’s helicase activity [17]; strong folds may occlude initiation sites such as the Shine–Dalgarno sequence [18]; folding mediates the mRNA’s susceptibility to degradation [19] or it modulates ribosome velocity and efficient queuing [20] (for a broader critique of the queuing/ramp hypothesis see [21–26]). Native *E. coli* genes also tend to be A enriched and G poor at 5′ ends, with synonymous sites unusually conserved [15].

In addition to A enrichment and G avoidance, NGG codons are found to be suppressive when in the 5′ end [27]. That GGN and GNG codons do not have similar effects [27] suggests that this effect is not dependent on nucleotide content and hence probably not mediated by RNA stability effects [27]. It was subsequently shown that this effect was a result of aborted translation owing to peptidyl-tRNA drop-off at NGG [28, 29]. While the effects of nucleotide composition on RNA stability need not be assayed on a codon by codon basis, the codon is both a convenient unit and, owing to these known translational effects, a necessary metric in some contexts. Here then we consider the codon as the unit of interest. This is also very convenient for transgene design as we are often constrained to preserve amino acid content, being free only to choose between synonymous codons [30–33].

In this context, there exists an unresolved exception [34]. In the first massive transgene study to consider the effects of 5′ codon composition, Goodman *et al.* [2] in analysing >14 000 constructs differing at 5′ ends, calculate for each codon the degree to which it is over-represented, compared to it synonyms, in the most effective transgenes (those with the highest protein output controlling for construct design and plasmid copy number). For each codon they could thus provide a single figure, one that is comparable between codons of different amino acids (see their Fig. 2 [2]; for experiment description see 'Materials and methods'). While they report the influence of third site A content and then point to the role of RNA stability (independent of other codon biasing effects), the codon with the greatest association with high output transgenes was, by some margin, AGG from the 6-fold arginine block. As this should be suppressive, according to the NGG rule, and should be associated with higher not lower stability, according to the G avoidance rule, this result is paradoxical. Additionally, two of three NGG codons were reported as boosting of protein levels relative to synonyms. Osterman *et al.* [14] have further highlighted that, as the Shine–Dalgarno sequence, that functions 4–6 bases 5′ of the initiating start codon, is AGGAGGU in *E. coli*, AGG within the first 10 codons may contribute to a distracting Shine–Dalgarno mimic sequence that could also suppress translation. This renders AGG’s apparent translational boosting effects yet more enigmatic.

Under the notion that one should “*Treasure your exceptions*” [1] we seek to understand this apparent anomaly. Does this unexpected result point to an underappreciated aspect of 5′ domain biology, potentially one important to transgene design? The NGG suppression is particular to codons 2–5 [27]. Might AGG have counteracting boosting effects, perhaps outside of this narrow domain? It was previously conjectured [35] that enrichment of 5′ ends in *E. coli* with non-optimal (“rare”) codons may be a necessary feature to enable orderly initiation and ribosomal queuing (see also [36]). This model assumes that rare codons slow ribosomes, a common assumption but one with a complex evidential history (see supplement of [37], as well as [23–25]). That AGG is the rarest codon in *E. coli* [38] and the most depauperate in the core region of highly expressed genes [34] (i.e. the most non-optimal) then may be of considerable relevance [34]. *Prima facie* the extreme association between 5′ AGG and protein production and its extreme non-optimality may indeed be considered as support for the hypothesis that slow to translate 5′ codons are important for protein production.

The role of rare codons in 5′ ends is, however, contentious. A core problem is that, as in *E. coli* rare/non-optimal codons tend to be A-ending, any other force causing high A content, such as selection for low stability, would by necessity lead to enrichment of translationally non-optimal codons. This renders the influence of both parameters hard to unravel [5]. One analysis reports that codon adaptation is negatively correlated with 5′ ribosomal occupancy and, importantly, that this effect is independent of other factors potentially affecting orderly initiation, including low RNA stability [20]. By contrast, Goodman *et al.* construct transgenes enriched for rare codons at the 5′ end and show that, while these have the greatest protein output, codon optimality (or lack thereof) is not predictive of protein level when controlling for RNA stability effects [2]. Bentele *et al.* likewise report that any enrichment of rare codons in native genes is parsimoniously explained as an epiphenomenon of RNA stability effects mediated by nucleotide skews [21]. They find the 5′ enrichment for rare codons to be particular to those ending A/T and show that there are no consistent effects of protein production when RNA stability is controlled but codon adaptation is altered. As a direct test of the role of tRNA availability, Osterman *et al.* [14] removed three of four tRNAs that decode CGU, CGC, and CGA, but observed no effect of these codons’ influence on biosynthesis. Allert *et al.* [5] also rationalise that if what they term codon non-compliance (meaning usage of rare codons) is a force, then they expect to see in AT rich genomes a preference for GC rich 5′ ends, but do not observe this. Partially in response to some of the above evidence, the original advocates of the codon bias slowing hypothesis, argued that transgene evidence is not reflective of native genes as they represent more extreme RNA stability than seen natively [39] (see also [40]). Moreover, they suggest that absolute rarity *per se* is not relevant, but rather that the 5′ end needs merely to have lower codon adaptation to the tRNA pool than the following region [39].

Before we consider biological hypotheses, we here first seek to exclude explanations that might instead consider the AGG effect an artefact. First, Goodman *et al.* [2] employ an unusual metric of protein output that they call Prot.FCC. This compares between constructs that have the same amino acid content and same 5′ UTR content, and generates a protein level compared to a constitutively expressed reporter (so controlling for plasmid copy number) to find a likelihood score that they treat as comparable between constructs that differ in amino acid content and 5′ UTRs. If we instead consider the more commonplace statistic, protein titre per RNA, across all constructs, redefine log odds ratios, does AGG still have the highest score? We find that it does.

A second possibility is that AGG’s enrichment in high productivity constructs may be particular to the constructs that Goodman *et al.* [2] employ. To address this, we ask whether AGG’s unexpected score is repeatable. To this end, we derive the log odds ratio enrichment profile of codons in tHEGs in the 244 000 transgene set of Cambray *et al.* [7] (for experimental details see 'Materials and methods'). In this dataset, we find NGG are suppressive and AGG is enriched in the low output constructs i.e. the Goodman result is not replicated.

More generally, across all codons we unexpectedly observe only limited concordance between the two massive transgene datasets. While variation in experimental protocols will in no doubt cause some variation, we show that the discrepancies are largely owing to what we term passenger effects: non-random distributions of codons, particularly within the Goodman data, that undermines, to a nontrivial degree, the ability to make causal inferences about the role of each codon in modulating protein level. It explains AGG’s outlier status, the Goodman constructs with AGG being enriched for non-AGG codons that themselves promote protein level and low RNA stability even though AGG itself does not. These results underscore the importance of proper randomization of constructs if one wishes to make unbiased inferences concerning the underlying biology.

While the above result explains the AGG anomaly, it doesn’t address the more general question of whether lower 5′ codon optimality predicts protein level. Thus, we then consider what features of the first 10 codons do predict protein per transcript in the Cambray data. We find, unexpectedly, that high, not low, codon adaptation is weakly associated with higher output. This contrasts with prior results that suggest that codon adaptation to the tRNA pool in the 5′ end is not predictive of protein level when allowing for covariates (RNA stability) [2, 21], and not obviously consistent with the view that rare codons are needed for orderly initiation [20, 35].

As Goodman's data appears *prima facie* to be biased, we then scrutinize results that have been derived from analysis of this dataset. Specifically, and cognizant that transgene results need not translate to insights into native genes [39], we ask whether enrichment trends at 5′ ends of native highly expressed genes (nHEGs) are discrepant from those associated with high transgene expression, as recently observed [34], and whether 5′ enrichment trends compared to gene cores are predictive of transgene enrichment trends [34]. For both, we replace the Goodman data with the Cambray data. We find the prior results to be qualitatively upheld.

## Materials and methods

### The experimental datasets

Goodman *et al.* [2] conducted the first large-scale transgene study to examine the effects of 5′ codon composition, selecting 137 endogenous essential *E. coli* genes, designing each into transgene constructs. These constructs were expressed using one of two promoters and one of four ribosome binding sites (three of varying strengths and the wild type). For each promoter/ribosome binding site combination, for each of the 137 genes, 13 different constructs were generated differing at synonymous sites in the first 10 codons after the start codon. This generated a total of over 14 000 different synthetic sequences. Each sequence was positioned upstream of super-folder Green Fluorescent Protein (sfGFP) on a plasmid that constitutively co-expressed mCherry, allowing for normalisation of expression levels. The experiment was conducted using *E. coli* MG1655. DNA and RNA levels were quantified using DNA sequencing (DNASeq) and RNASeq, while protein levels were measured using FlowSeq. They report various measures of protein level: Prot.FCC considers protein level compared to the constitutive mCherry and considers the magnitude of differences between the 13 directly comparable constructs; Trans.FCC is the same but considers protein per RNA. Trans, the metric we dominantly employ, considers protein per RNA without between-construct comparison.

Cambray *et al.* [7] generated a massive library of synthetic sequences starting by calculating values of 8 sequence properties (including codon usage bias, AT content, RNA secondary structure, and codon ramp bottleneck position and strength) previously reported to influence expression for all 3990 protein coding genes annotated in a reference *E. coli* genome. They examined these properties in relation to previously published mRNA and protein abundance data to find the relevant physiological ranges, and assigned two or three discrete levels to each property (resulting in 1458 combinations). For each combination, they then generate three variants (with 1–4 synonymous nucleotide changes), and for each variant used full factorial design to derive 56 fully independent mutational series. This generated a library of over 244 000 synthetic sequences. Each sequence was inserted via a cleavable *N*-terminal translational fusion to an sfGFP reporter to allow for standardised protein measurement in *E. coli* MDS42. Fluorescence was analysed using FACS (fluorescence-activated cell sorting) and high-throughput sequencing (DNAseq and RNAseq). To quantify translation efficiency, Cambray *et al.* measured protein production under two different conditions: PNI (normal translation initiation), where translation occurs naturally, and PFI (facilitated translation initiation), where an inducible bicistronic controller increases ribosome accessibility to the start codon. We use both these protein measures, along with their RNA_SS_ metric for RNA levels. They also provide cell growth rates under normal (WNI) and facilitated (WFI) translation initiation.

Osterman *et al.* [14] constructed a library of variants with a randomised 30-nucleotide region immediately downstream of the start codon. The sequences were designed to investigate how codon identity, Shine–Dalgarno-like motifs, and RNA secondary structure influence translation efficiency. After filtering out low-abundance variants and sequencing errors, over 30 000 unique sequences were retained for analysis. Each of the synthetic sequences was inserted into a dual-fluorescent reporter construct expressing Cerulean fluorescent protein (CER), with red fluorescent protein (RFP) as an internal control for normalisation. The library was introduced into *E. coli* BW25113, and cells were grown in LB or M9 minimal medium. FlowSeq was used to sort cells into five translation efficiency fractions (TEFs) based on the CER/RFP fluorescence ratio. DNA was extracted from each fraction, and high-throughput sequencing (Illumina MiSeq) was used to determine the frequency of each variant across the TEFs. As Osterman *et al.* don’t provide raw RNA and protein levels, we use TEF as a measure of transgene expression.

### Calculating log odds ratio vectors

To provide a fair comparison across different datasets, we use a method that evaluates codon enrichment within each synonymous block in the upper or lower quartile of a given metric (e.g. highly v lowly expressed genes). This approach allows for differences in amino acid usage, particularly important in transgene design where amino acid changes are commonly undesirable, and provides a metric directly comparable to that employed by Goodman *et al.* [2].

Fifty-nine element log odds ratio vectors were used as measures of codon enrichment relative to other synonyms, comparing between sequences based on a given feature/condition. Considering in turn all codons coding for degenerate amino acids as the focal codon, log odds ratios were calculated with the given formula:


\begin{eqnarray*}
&& log\;odds\;ratio = \\ && ln\frac{{count\;of\;focal\;codo{n_{\;condition\;1}}/count\;of\;synonymn{s_{\;condition\;1}}}}{{count\;of\;focal\;codo{n_{\;condition\;2}}/count\;of\;synonym{s_{\;condition\;2}}}}
\end{eqnarray*}

In all cases, condition 1 is the upper quartile of the relevant metric and condition 2 the lower quartile of the same metric. This generates a vector of log odds ratios for the 59 codons (excluding stop codons or those without synonyms).

To account for those instances where a codon is not present in either, or both, conditions 1 and 2, we use the modified Haldane–Anscombe method for zero-cell corrections [41]. This involves an addition of 0.5 to the count of all four components of the above formula, if only one or some of the components, but not all, are zero. If instead all four components are zero, we take this to reflect absence of data and attribute ‘NA’ to the log odds ratio score for all codons encoding that amino acid.

The method can be applied to any metric (for the resulting vectors see Supplementary Table S1). We consider various protein level metrics applied to the full 5′ CDS span (codon 2 to codon 11, where codon 1 is the start codon): protein level per RNA employing production under normal translation initiation (PNI/RNA_SS_) and under facilitated translation initiation (PFI/RNA_SS_) for the Cambray data; Prot.FCC for the Goodman data, a metric of relative protein abundance that compares between 13 variants that share the same ribosome binding site, promoter and 5′ protein sequence, i.e. that differ only in their synonymous site content; Trans also provided by Goodman *et al.* [2] reflecting protein per RNA not controlling for construct features; Trans.FCC the same as Trans but controlling for construct features. For native *E. coli* genes, protein abundance was retrieved from PaxDB [42] and RNA measures from a compendium of RNA-seq datasets [43]. We considered native RNA data in four different ways: mean of all measures with no data treated as zero, mean of all measures with no data treated as NA, median of all measures with no data treated as zero, median of all measures with no data treated as NA. For Cambray's PNI/RNA_SS_ metric and Goodman’s Prot.FCC and Trans, we also generate log odds vectors by 5′ position (+2 to +11, where the start codon is at position +1) of each sequence.

We also consider three different 5′ stability metrics for the Cambray constructs, namely our calculation using the ViennaRNA R package [44] for the 5′ ends and their values supplied for −30 to +30 bp (the 5′ UTR section being identical in all cases) and 0 to +60 bp (i.e. their metrics “ds.utrCdsStructureMFE” and “ds.fivepCdsStructureMFE” respectively). We considered the three stability metrics provided in the Goodman data (i.e. their metrics “dG”, “dG.noutr”, “dG.unif”) and our own estimation of the 5′ ends (with ViennaRNA R package [44]). We selected the pairing that maximized the log odds ratio correlations (Supplementary Table S2) between the Goodman and Cambray 59 element log odds ratio vectors so as to minimize disagreement owing to methodological variation.

We also perform the log odds ratios analysis on the 30 000 transgene dataset of Osterman *et al.* [14] so as to compare results when these constructs are expressed in cells grown in LB and M9 minimal media (for experiment details see above). Since they do not provide raw RNA or protein measurements but only a course-grained integer-based TEF scale (ranging 1–5), we adapt our approach accordingly. To classify constructs into high and low expression groups, we respectively select those in the highest and lowest TEFs and then randomly subsample from the adjacent fraction to ensure that each group comprises 25% of the total constructs. For LB medium, we use TEF 1 plus subsampled TEF 2 for low expression and TEF 5, TEF 4 plus subsampled TEF 3 for high expression. For M9 minimal medium, we use TEF 1 plus subsampled TEF 2 for low expression and TEF 5 plus subsampled TEF 4 for high expression. We then compute log odds ratios for each codon, compared to its synonyms, as explained above.

To assess the variability that we would expect to arise when repeating an experiment under the same conditions, for Osterman data, we repeat the analysis applying bootstrapping with replacement when sampling constructs from each TEF group. We thus repeat the log odds ratio calculations 100 times, each time resampling both the highly and lowly expressed construct groups. This provides a distribution of log odds ratios for each codon rather than a single estimate.

We obtained input data from the respective supplementary files: Supplementary Data 15 of Cambray *et al.* [7], Supplementary Table S1 of Goodman *et al.* [2], Supplementary Table S1 (for full 5′ domain) from Lewin *et al.* [34] and Supplementary Table S1 (for LB medium) and Supplementary Table S4 (for M9 minimal medium) from Osterman *et al.* [14].

### Relative importance of regressors in linear models (relaimpo) analysis

Relative Importance of Regressors in Linear Models (relaimpo) is useful to attribute explanatory strength to each predictor variables allowing for covariance between each. All relaimpo analyses were conducted using the R package relaimpo [45].

We firstly use a relaimpo analysis to assess relative position influence on protein levels. We employ the Cambray data to determine the relative impact of sequence GC content of each codon in position +2 to +11 on predicting PNI/RNA_SS_ (model: PNI/RNA_SS_ ∼ GC_codon_2 + GC_codon_3, etc.). We also employ it to determine the potential predictors on protein per RNA, also considering the Cambray data. Here the model is: Protein (either PNI or PFI)/RNA_SS_ ∼ stab30 + stab60 + CAI + delta CAI + NGG + Pro + Pos + GC, where stab30 is the stability of the construct from −30 to +30, likewise stab60 for 0 to +60 bp (both metrics reported by Cambray *et al.*), Codon Adaptation Index (CAI) is the log odds ratio for each codon reflecting codon enrichment in the core of highly expressed native genes, assayed using protein abundance (see [34]), delta CAI is the difference between the CAI metric for 5′ ends (taken to be the first 10 codons) and that of the rest of the manipulated codons (up until 30 codons after the start codon), NGG is the density of NGG codons in the first 6 codons (where N can be any of the four nucleotides), Pro is proline density in the first 10 codons, Pos is the density of positively charged amino acids, and GC is the G + C nucleotide content of the first 10 codons following the start codon.

### Statistics to calculate codon concordance

For each amino acid at each codon position (+2 to +11) we determine, for both the Goodman and Cambray data, the codon that has the highest log odds ratio. This gives an 18 amino acids x 10 positions matrix (we exclude non-degenerate amino acids). These two matrices should then present the preferred codon for that amino acid at that position in a synthetic transgene. To determine whether the two datasets agree, for each position and each amino acid, we score +1 if the two agreed and 0 if not. For each amino acid, we then determine the probability of the observed degree of concordance employing a binomial test (binom.test in R), where observed successes is the sum of the amino acid row (i.e. the number of + 1s), total number of trials is the number of non-NA elements for each amino acid comparison, and expected number is derived from 1/degeneracy. To determine an overall statistic, we repeat the same with the total number of agreements being successes across all positions and all amino acids, number of trials being the total number of non-NA elements and p (the weighted mean [1/degeneracy]) weights being given by the number of non-NA entries for each amino acid.

### Testing for passenger effects

We define passenger effects as any distortion in the log odds ratio of a given codon owing to that codon being non-randomly distributed with respect to all other codons in a given set of constructs. To determine the extent of passenger effects for any given focal codon in any set of constructs, one must then determine the effects of the non-focal codons in the constructs in which the focal codon is found. To this end, one needs to predefine a metric of the effects of the other codons. This can be done by using the observed log odds ratios of every codon within the same dataset or by usage of a log odds ratio score from a dataset that *a priori* has no or limited passenger effects.

Algorithmically, we ask the following. First, we identify those constructs that contain the focal codon. For each such construct, we then determine the mean log odds ratio of all the non-focal codons within that same construct. Repeating for all constructs containing the focal codon, we determine the mean of the means of the log odds ratios. The sample size (N) we define as the number of constructs with at least one example of the focal codon rather than the number of focal codons in the constructs as this would lead to reusage of the data from constructs with more of the focal codon i.e. pseudoreplication. We determine the standard deviation (SD) between the mean log odds ratio of the non-focal codons per construct. Standard error of the mean we then derive as SD/sqrt(N). An advantage of this metric is that *a priori*, given the nature of log odds ratios, the null mean of means should be zero for a perfectly randomized construct library. Any codon disproportionately in construct contexts that reduces protein level will have a negative score, while codons whose construct context artificially boosts their score will have a mean of mean log odds ratio greater than zero.

For there to be passenger effects, there must be non-random associations between codons across constructs. To determine whether codons are non-randomly distributed (without reference to log odds ratios), we consider for each codon the extent to which the other codons are differentially in, or not in, the same constructs. For each codon, we consider those constructs containing it and ask about the count of each non-focal codon in the same constructs. This gives us a count of each pairing [focal codon, non-focal codon in same constructs]. We determine the expected value for each pair count by considering the total number of all non-focal codons in the same constructs (those with the focal codon) and multiplying this by the frequency of each non-focal codon (amongst the non-focal codons) in the dataset as a whole. With 61 focal codons and 60 non-focal ones, this generates 3660 comparisons between observed and expected values. From these we derive two metrics. First, we calculate the sum chi squared value. As chi squared is sensitive to total counts, to provide fair comparison between Cambray and Goodman constructs, we randomly subsample 14 234 sequences from the Cambray set (the same number as in Goodman data). Doing this 1000 times gives a distribution of sum chi squared values for the Cambray set that is directly comparable to the Goodman set. Secondly, to provide a metric of the extent to which there is non-random association between codons at the level of the codon (rather than the constructs as a whole), we determine for each codon the mean value of (|O-E|)/E across all the codon-codon pairs for that focal codon, O being the Observed codon-pair count, E the Expected, as defined above. Unlike the chi squared statistic, this metric should be largely unaffected by differences in counts between focal codons. We again subsample from the Cambray data and present mean and SD of the mean (|O-E|)/E amongst the 1000 randomised subsamples.

### Sequence retrieval for cross-species analysis

We also perform a codon enrichment log odds ratio analysis as described above, but comparing 5′ codon enrichment (as condition 1) to enrichment in the gene core (as condition 2), across 1355 bacterial species. All sequences required for this were obtained from RefSeq NCBI [46]. See Supplementary Table S3 for the full list of accessions for the analysed species.

### Script availability

All input data and downstream scripts, both processing and figure/statistical analyses are available at https://doi.org/10.5281/zenodo.15084715.

## Results

### AGG’s association with high protein output is independent of the definition of “output”

**Figure 1.**
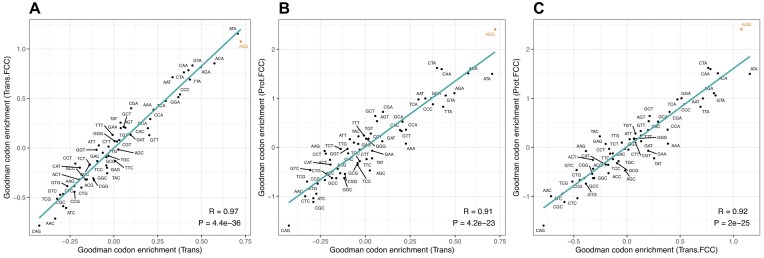
Comparison of log odds ratios for each codon as a function of the metric of “protein level” in the Goodman *et al.* data. Log odds ratios represent a measure of codon enrichment in the upper quartile of protein level. We employ three metrics of protein level: Prot.FCC, a metric of relative protein abundance employed predominantly by Goodman *et al.* that compares between 13 variants that share the same 5′ UTR and amino acid content, and compares with the level of a constitutive reporter; Trans, a metric of protein per RNA; Trans.FCC, the Trans metric compared between 13 variants that share the same 5′ UTR and amino acid content, and with the level of a constitutive reporter. The three panels show each pairwise comparison in log odds ratios between the three protein level metrics with (**A**). comparing Trans v Trans.FCC, (**B**). comparing Trans v Prot.FCC, and (**C**). comparing Trans.FCC v Prot.FCC. In all panels, AGG is highlighted, and respective Spearman correlation and *P*-values are displayed on each plot.

AGG’s strong association with high Prot.FCC scores [2, 34] may be a consequence of the unusual nature of the Prot.FCC metric. We thus first ask whether the metric matters. To address this, we consider log odds ratios for all codons but now employ two different metrics: the more classical protein per RNA (Goodman's “Trans” metric) and this metric compared between the same constructs (“Trans.FCC”). We find that AGG has an unexpectedly high log odds ratio no matter the metric (Fig. 1, Supplementary Table S1). For the protein per RNA metric, AGG has the highest score and for the same normalised within constructs it has the second highest. Given that the Trans metric is both comparable with data from other experiments and controls for RNA level, we henceforth consider protein by RNA as the focal metric. We also consider Prot.FCC to relate back to prior results.

### AGG’s association with high protein output is not replicated

Is AGG’s association with high transgene output replicated in alternative transgene datasets? More generally, do transgene datasets agree on synonymous codon enrichment trends at genic 5′ ends that are associated with high output? To address this, we consider the 244 000 transgene dataset of Cambray *et al.* [7]. To ask whether the two transgene datasets broadly agree on the codon enrichment patterns, we compare patterns of enrichment in the most highly and most lowly expressed transgenes (tHEGs v tLEGs) within each set. We compare the Goodman Trans metric with Cambray’s data on protein level normalised to the RNA level, rendering the two metrics directly comparable.

**Figure 2.**
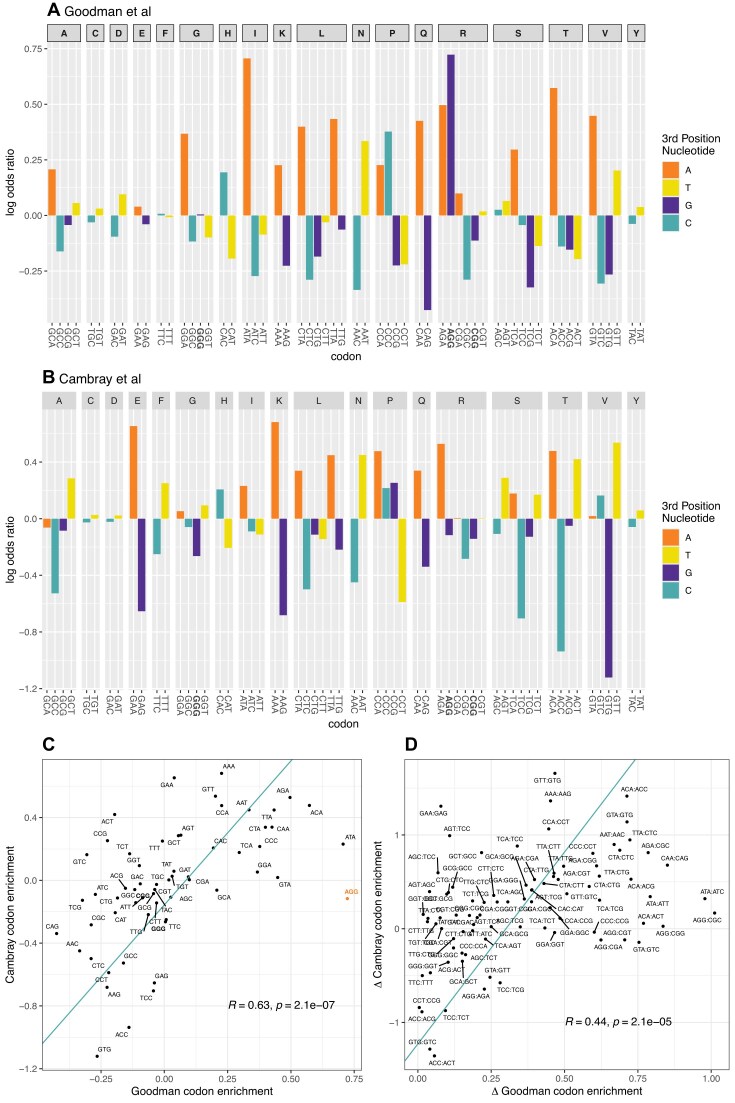
Cambray v Goodman overall 5′ codon preferences in highly v lowly expressed transgenes (all 10 positions following the start codon). Log odds ratios for enrichment in the top quartile by expression for each codon within a synonymous codon block for (**A**). Goodman data (using Trans, i.e. protein per RNA) and (**B**). Cambray data (protein per RNA). (**C**). The *x*-axis is the log odds ratio for the codon being enriched at the 5′ ends in Goodman transgenes with high protein per RNA compared to low protein per RNA, the *y*-axis is for Cambray transgenes. Each data point is labelled as the codon it represents, AGG is highlighted. For panels A, B, and C, see NGG codons in bold. (**D**). As for panel C, but comparing all pairwise combinations of synonymous codons (i.e. within the same codon block: *N* = 87). The pairwise differences are oriented such that, on the *x*-axis, the codon with the lower value of the log odds ratio has its value subtracted from that of the higher value. The orientation is preserved for the *y*-axis. Each point is labelled by the oriented codon pair (first codon in the pair has the higher *x*-axis value, as seen in panel C). For both C and D, Principal Components Analysis (PCA) was used to fit an orthogonal regression line, and the Spearman’s correlation coefficient and *P*-value are provided within the plots.

In the first instance, we consider usage across all 10 codons following the start codon. AGG has a negative log odds score (i.e. is depauperate) in the 5′ end of tHEGs in the Cambray data (Fig. 2A). More generally, unlike the Goodman data (using both Prot.FCC (Supplementary Fig. S1) and Trans (Fig. 2B)) in which two of three NGG codons have a positive log odds score when considered across the full 5′ end, all NGG codons have a negative score in Cambray data (Fig. 2A), commensurate with a suppressive effect [27]. The Goodman result is thus not replicated. More generally, the codon enrichment patterns when using protein by RNA for each dataset broadly agree (Fig. 2C, R = 0.63, *P*= 2.1 × 10^−7^). We can repeat the same analyses but this time controlling for synonymous block structure. This way we ask whether, within each codon block, in any given pairwise comparison, the codon with the higher log odds ratio in the Goodman tHEG v tLEG comparison also has a higher log odds ratio in the Cambray comparison. This controls for the fact that within two-fold codon blocks there is non-independence of data as log odds of codon 2 is the negative of the log odds of codon 1. We find the agreement in codon enrichment patterns to also be significant when we compare between synonymous codons within a block if employing Trans (Fig. 2D, R = 0.44, *P*= 1.7 × 10^−5^), but not with Prot.FCC (Supplementary Fig. S1D, R = 0.19, *P*= 0.071). As both experiments were asking the same question – what features of 5′ ends determine protein level – agreement is to be expected. What is perhaps more curious is that two experiments disagree to a considerable degree even though the metrics are the same. Indeed, comparing protein by RNA metrics within synonymous blocks (Fig. 2D), over 80% of the variation is unexplained (Spearman correlation 0.44).

### Different transgene experiments weakly concur on codon enrichment trends in tHEGs at any given 5′ position

Given that NGG codons are thought to be suppressive but only at certain locations [27], it is also valuable to ask whether there are effects by codon position. For transgene design, this is of utility if one wishes to specify which codons to use for any given amino acid at any given position within the 5′ domain [47]. To address this, we determine, for each position following the start codon (up to codon 11), a log odds ratio for every codon (within a synonymous block). We take this to reflect its relative enrichment in the most tHEGs, for each of the two studies. We ask for each amino acid and each codon position (+2 to + 11), which codon has the highest log odds ratio. As regards arginine, in the Cambray data, AGA is the optimal codon at positions 2, 3, 5, 6, and 8–10 (Supplementary Fig. S2). Given the A enrichment rule, this is as expected. At position 7, where previously AGG was reported as not being influential [27], AGG is reported as the optimal codon (Supplementary Fig. S2). Goodman's original Prot.FCC data suggest that AGG should be optimal at positions 4–11, excluding 6 (Supplementary Fig. S3). Using the Trans metric for the Goodman *et al.* data, AGG is reported as the most protein promoting at positions 7, and 9–11 (Supplementary Fig. S2).

**Figure 3.**
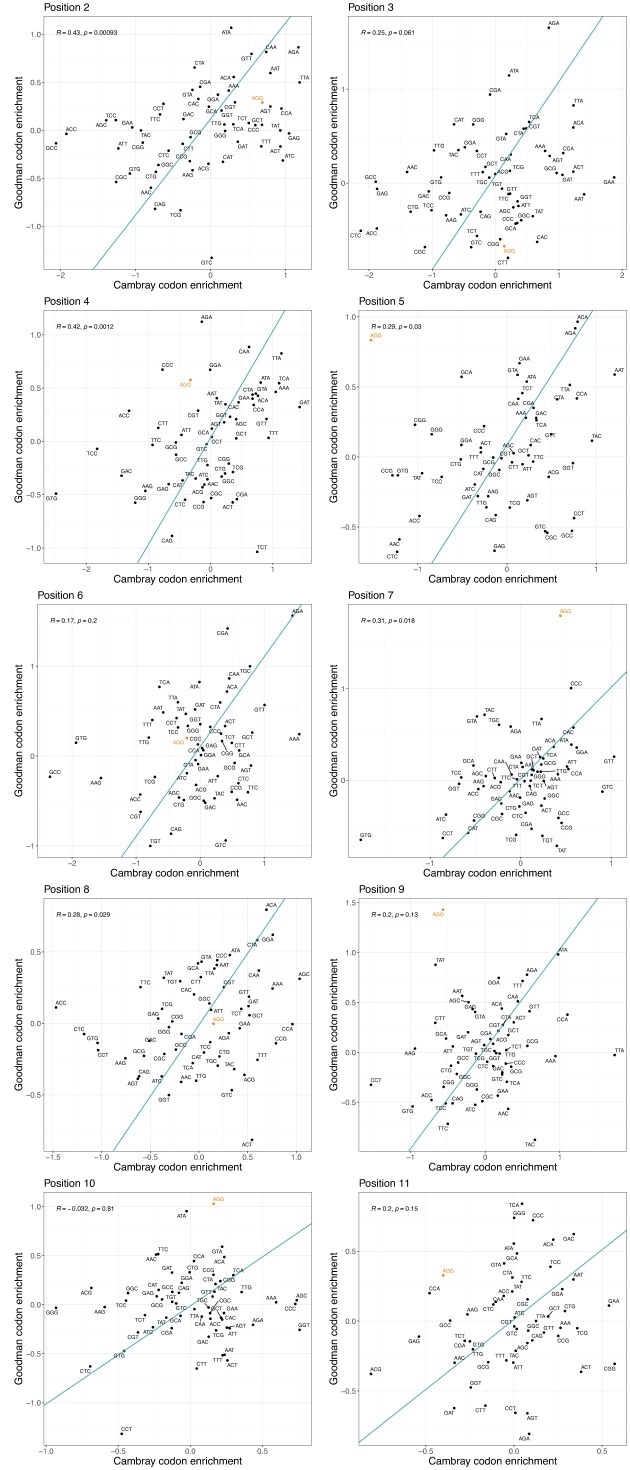
Cambray v Goodman 5′ codon preferences in highly v lowly expressed transgenes broken down by position. Each plot is as in Fig. 2C, but in this instance, broken down by amino acid position after the start codon. We employ protein/RNA for both datasets. For all plots, regression lines represent a linear model and AGG is highlighted.

More generally, our approach allows to ask whether the two transgene datasets agree on which codons to employ for any amino acid at any 5′ position. Under a null of no greater similarity than chance, we expect none to be significantly correlated after multi-test correction. However, as in principle they should be providing the same information, we expect the two to be strongly positively correlated at all sites that are influential (and a relaimpo analysis [45] for Cambray data (Supplementary Fig. S4) indicates that all sites are influential). Of the analyses by position comparing Goodman Trans data with Cambray protein per RNA, positions 2, 4, 5, 7, and 8 are significantly correlated, with only 2 and 4 remaining significant after Bonferroni correction (i.e. raw *P* < 0.05/10; Fig. 3).

**Figure 4.**
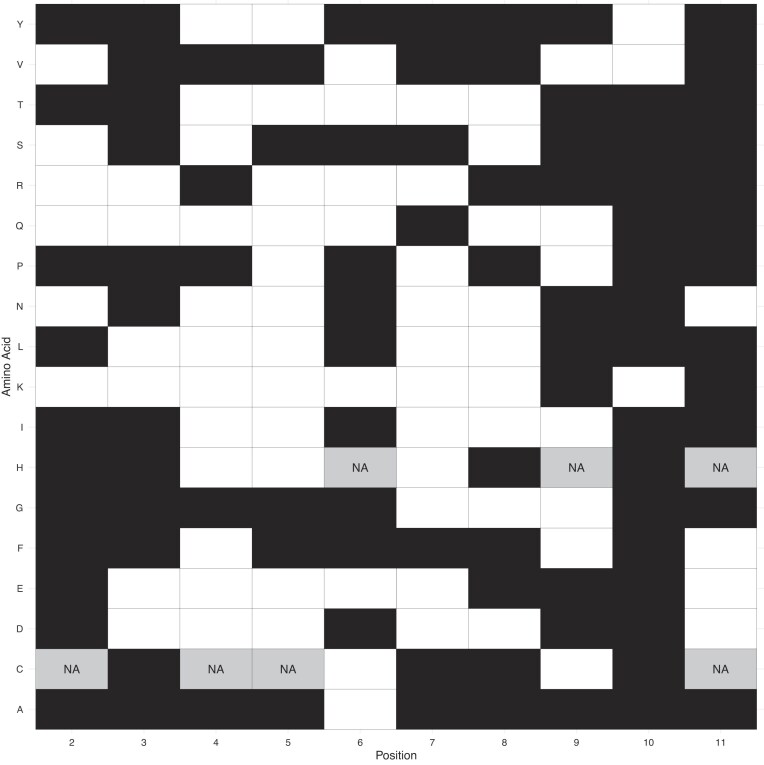
Agreement between Cambray and Goodman 5′ codon preferences in highly v lowly expressed transgenes broken down by position, by amino acid. For both datasets, the metric for transgene expression is considered as protein per RNA. For a given amino acid (see all listed vertically on the left), the position where the codon with the highest log odds ratio is the same in the two datasets is illustrated in white. Those in disagreement in black. NA is called when any given amino acid is unrepresented in either construct at the given position. For the codons nominated by the two analyses see Supplementary Fig. S2.

From both sets of transgene data we can then isolate the one codon for each amino acid at each position that is most enriched in tHEGs. We find that the two analyses do not, for the most part, agree on which this codon is (Fig. 4). We treat each row of the tile plot as a binomial plot (x = number of white tiles, N = number not NA, *p*= 1/degeneracy, see 'Materials and methods') and find a weighted mean null to derive an overall expected number of agreements under a null of no more agreement that expected by chance. The overall concordance is marginally more than null (binomial test, 78 agreements from 173 trials, null *p*= 0.36, *P*= 0.014; Fig. 4).

Using the original Prot.FCC metric as employed by Goodman makes little to no difference to the above. We find significant positive correlations at codon positions 2–5, and for all of them to also be significant after Bonferroni correction, but positions 3 and 5 only weakly (Supplementary Fig. S5). As regards agreement about the best codon for any amino acid at any position, using as above mean null concordance and doing one binomial test for the tiles overall, the *P*-value is again marginally significant (binomial test, 75 successes from 173 trials with weight mean null expectation *p*= 0.36: *P*= 0.048, two tailed, 62 expected under the null of no agreement; Supplementary Fig. S6).

### Evidence that passenger effects distort Goodman data

The above disagreements between the Cambray and Goodman data, which persist no matter how we treat the Goodman data, suggest that AGG’s enrichment in tHEGs (in the Goodman data) is more likely to reflect a difference between experiments rather than reflecting important underlying biology. One possible explanation for AGG’s anomalous log odds ratio in the Goodman data, and the discrepancies between the datasets, is a non-random association between codons within the examined constructs.

Imagine, for example, that AGG is not boosting of protein level, nor reduces 5′ stability, but is present (by chance or design) more often in constructs that have other codons that are causative of high expression than elsewhere. If so, AGG would be allocated a high log odds ratio as it is disproportionately seen in tHEGs as opposed to tLEGs. Such a model bears resemblance to concepts of linkage disequilibrium, hitchhiking and passenger mutations in cancer, all of which see the fate of one entity as being owing to the effects of variation in the causative physical neighbours with which it is non-randomly associated. We thus refer to the non-random codon distribution hypothesis as a passenger effect. We define passenger effects as any distortion in the log odds ratio of a given codon owing to that codon being non-randomly distributed with respect to all other codons in a given set of constructs. Avoidance of such effects is indeed the motivation behind full randomization protocols [7].

**Figure 5.**
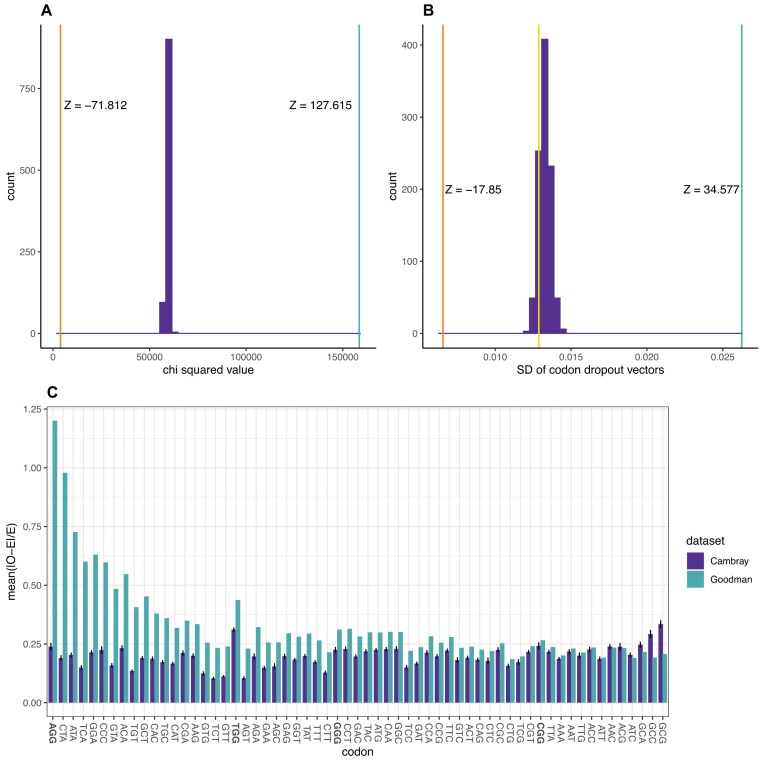
Evidence of passenger effects affecting Goodman and Cambray datasets. (**A**). Chi squared values of observed co-occurrences between a focal codon and any given non focal codon under a null of random association. The histogram is the distribution of chi squared values calculated for each of the 1000 Cambray random subsampling of 14 234 sequences. The lower orange line indicates the 1% significance level with df = 3659. The higher light blue line is the sum chi squared value for the Goodman data. (**B**). Comparison of the variation (measured as SD) within the vectors of mean log odds ratios for flanking codons for each codon when present in a construct. The histogram represents the distribution of Cambray construct values when 1000 random sets of 14 234 sequences are extracted from that dataset. The vertical yellow line is the SD of the vector of mean of the log odds ratio when each codon’s neighbour are considered in the complete Cambray data (*N* = 232 841). The higher light blue line is the comparable vector for the Goodman constructs (with the Cambray log odds ratios applied). The lower orange line is the approximation to the null expectation derived by considering for each codon the mean of the Cambray log odds ratio vector without that focal codon (i.e. under the assumption that all codons flank all other codons at an equal rate). For a true randomized dataset with all codons used equally, the mean log odds ratio of codons in the same construct should be the mean of those in the log odds vector. The value is the SD of the null vector. The Z values represent the deviation between the lines in units of the SD of the distribution of (subsampled) data. (**C**). Mean (|O-E|)/E values as panel A for each codon separately. The codons on the *x*-axis are rank ordered by the difference between datasets (Goodman – Cambray). NGG codons are highlighted in bold. Note AGG is the leftmost codon, showing the highest degree of non-randomness in the neighbourhood in the Goodman dataset and the most difference to its value in the Cambray dataset. The Goodman constructs have higher non-randomness than the Cambray constructs in 55 of 61 by codon comparisons (binomial test, *P* = 5 × 10^−11^).

To first test for differences between any two sets of constructs for presence in passenger effects, we consider for each codon the extent to which the other codons are differentially in, or not in, the same constructs. For each focal codon (*N* = 61), we consider those constructs containing it and ask about the count of each non-focal codon in the same constructs. This gives us a count of each pairing [focal codon, non-focal codon in same constructs]. We determine the expected value for each pair count by considering the total number of all non-focal codons in the same constructs and multiplying this by the frequency (amongst the non-focal codons) in the dataset as a whole of each non-focal codon. With 61 focal codons and 60 non-focal ones, this generates 3660 comparisons between observed and expected values, for which we calculate the sum chi squared value. We can then compare the Cambray and Goodman constructs. However, as chi squared is sensitive to total counts, to provide fair comparison, we randomly subsample 14 234 sequences from the Cambray set (the same number as in Goodman data). Doing this 1000 times gives a distribution of sum chi squared values for the Cambray set that is directly comparable to the Goodman set (Fig. 5A). With 3659 degrees of freedom, we also determine the one percent significance level. This reveals much greater deviation from random for the Goodman constructs (Fig. 5A).

What effect does such non-randomness have on log odds ratio skews of focal codons and their neighbours? To determine this, we consider the variation in each dataset in the mean values of log odds ratios of the codons in the same constructs as any given focal codon. Our expectation is that a randomised set of constructs will have a low but non-zero variance of values. We thus ask whether there is any difference in the between-codon variability in mean log odds ratios of the codons flanking any given codon, and hence a difference in passenger effects.

To ascertain this, we reanalyse both the Goodman and the Cambray set of constructs. We calculate the mean log odds ratio of the codons flanking any focal codon in the constructs in which it is present. We repeat for each codon and generate, for each sample, a 59 element vector. In all cases, we employ the Cambray high-low quartile protein/RNA metric to determine the log odds ratio for any given codon. Variation within these vectors is measured as SD. To control for sample size differences, as above, we randomly extract 14 234 sequences from the Cambray set for each random subsampling. Repeating this 1000 times, we see no Cambray randomised dataset (i.e. the 59 element vector) with as high a variability as observed in the Goodman data using the Cambray log odds ratio values (Fig. 5B). *Prima facie* this indicates the two datasets are different in a manner that cannot be ascribed to variation owing to differences in number of sequences *per se* or the input log odds ratio scores (Z approximation = 34.6, *P* < 0.001).

The above treats the datasets en masse. We can also determine the extent to which any given codon is non-randomly associated with other codons. As above, we first determine the observed and expected values for each focal/non-focal codon pair within the constructs that feature the focal codon. To allow for differences in codon abundance (which would affect chi squared scores), we compute the absolute difference between the observed and expected values, and then divide this by the expected value (i.e. (|O-E|)/E). For each focal codon, we then calculate the mean of this statistic across its 60 focal v non-focal pairs. We again subsample 14 234 sequences from the Cambray dataset 1000 times to eliminate any net sample size bias. To visualize the results, we present the mean statistic for each codon, ordering them by the difference in their statistic between the Goodman and Cambray datasets, where a higher value indicates greater deviation from random in the Goodman set (Fig. 5C). We find that for 55 out of 61 codons, the Goodman dataset presents larger deviation (binomial test, *P* = 5 × 10^−11^ Fig. 5C). This reinforces the above result, namely that the Goodman constructs are more non-random than the Cambray constructs. Notably, we observe that AGG has both the highest degree of non-random association in the Goodman dataset and shows the greatest difference when compared to its value in the Cambray dataset (Fig. 5C). This strongly supports the hypothesis that AGG’s outlier status is, at least in part, a consequence of construct design and not of, for example, between-experiment variation (e.g. in media, growth conditions, strains employed etc.).

We conclude that the Goodman dataset has more acute passenger effects (Fig. 5), with AGG especially affected. On all metrics, the Cambray set is also distant from the randomized null expectation derived from its own log odds values (Fig. 5).

### Passenger effects explain much of the disagreement between datasets

Does the difference in passenger effects explain the differences between the Goodman and Cambray log odds ratios? To examine this possibility, we consider for each focal codon, in each dataset, the mean log odds ratios of all other codons in the constructs (first 10 codons after the start codon) that contain at least one of that focal codon but excluding the log odds ratio of the focal codon from these calculations (as above). We determine the mean log odds ratio for each such construct and then the mean of the means of all such constructs (i.e. mean for all the constructs containing the focal codon).

**Figure 6.**
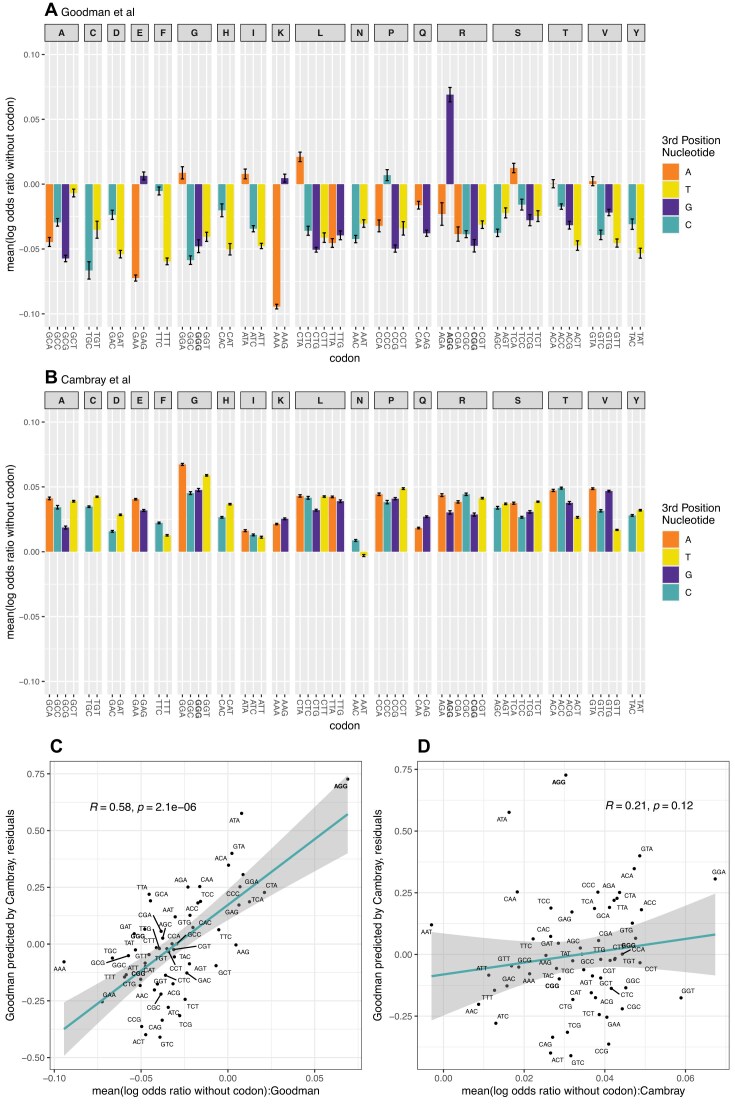
The relationship between non-random codon allocation to constructs and deviation between datasets using protein/RNA log odds ratios from Cambray data. For the Goodman, using Trans i.e. protein per RNA (**A**), and Cambray, using protein per RNA (**B**), datasets we considered for each construct containing a given codon the mean log odds ratios of the other codons in the same construct. We calculate the mean of these values for each construct, and then the mean of means for all constructs containing the focal codon. Here we use the log odds ratios derived from the Cambray dataset. The null is a mean of zero. As can be seen, the Goodman data have some highly deviant data points, notably AGG whose flanking codons have a highly positive log odds ratio. To consider whether these deviations explain the differences between Cambray and Goodman log odds ratio, we consider the regression between the two. We then determine whether the residuals from this regression are predicted by the mean log odds of the flanking codons in the Goodman data (**C**) and the Cambray data (**D**). Statistics in C and D are Spearman rank correlations. Error bars in panels A and B are standard error of the mean (SEM). NGG codons are highlighted in bold.

As *a priori*, we consider there to be a problem with the Goodman derived log odds ratios (AGG being unexpectedly high) we should not employ Goodman log odds ratios to determine the mean log odds of the codons sharing the same constructs: we should not employ AGG’s high log odds ratio when determining the mean log odds ratio of the Goodman constructs containing AGG when AGG is not the focal codon of interest. A preferable approach then is to calculate the mean log odds ratio of the non-focal codons (sharing constructs with the focal codon) using the Cambray log odds ratios for analysis of both the Goodman (Fig. 6A) and Cambray (Fig. 6B) constructs.

Importantly, AGG is again an outlier in the Goodman data (Fig. 6A). This indicates that AGG tends to be found in constructs that promote protein production, even if AGG does not. Conversely, AAA, which in the Cambray data is the highest ranked in terms of protein output per RNA, is flanked by suppressive codons in the Goodman constructs, potentially explaining why it isn’t more highly ranked when it should be the highest according to the A-rich rule.

To consider this sort of effect more generally we consider the regression of Goodman log odds ratios (using the Trans metric) when predicted by the Cambray values (also using protein per RNA). We then take for each codon the residual from the regression line, considering this a measure of the disagreement between the two datasets. We observe a strong relationship between the residuals and mean log odds of non-focal codons in the Goodman data (Fig. 6C: R = 0.58, *P*= 2 × 10^−6^) with an approximately linear trend. Indeed, between 34% (Spearman correlation = 0.58) and 45% (Pearson correlation = 0.67) of the difference between the Cambray and Goodman data (i.e. the residuals) is explained by passenger effects affecting the Goodman constructs. Using Prot.FCC as the measure for the Goodman data (Supplementary Fig. S7), these numbers increase to between 45% (Spearman correlation = 0.67) and 60% (Pearson correlation = 0.77). Any passenger effects in the Cambray data do not predict the residuals (Fig. 6D: R = 0.21, *P*= 0.12, also seen using Prot.FCC (Supplementary Fig. S7D): R = 0.21, *P*= 0.11), supporting the hypothesis that the differences between the Goodman and Cambray data are largely owing to passenger effects in the Goodman constructs.

We conclude that around half of the difference between the two experiments in their estimation of the importance of each codon in promotion of protein level is explained by passenger effects affecting the Goodman constructs. *Prima facie* the Goodman log odds ratios for individual codons are systematically distorted by non-random codon allocation to constructs.

### Passenger effects distort codon-stability estimated relationships

If the passenger effects hypothesis is correct, then, given the importance of low 5′ stability to protein levels, we also expect the same trends to affect log odds ratios in the two datasets when, instead of a form of protein metric, we employ RNA stability metrics to determine the log odds ratios i.e. codon enrichment trends in upper and lower quartile by RNA stability. We consider four RNA stability measures for the Goodman data and three for the Cambray data. For both sets of constructs, one of the measures is our analysis of the same span of sequence using ViennaRNA R package [44]. To best minimize discrepancies between RNA stability measures, we then perform the 3 × 4 Cambray v Goodman comparisons of log odds ratios vectors and subsequently consider the one with the highest Spearman rho value in the correlation with protein/RNA log odds ratios (Supplementary Table S2). ViennaRNA R package [44] RNA stability measures applied to the 5′ ends alone provide the highest significant correlation (Supplementary Table S2) and so are employed. We then consider the residuals of the regression of the Goodman log odds ratios predicted by Cambray log odds ratios and ask whether the values seen for the passenger effects in each construct set predicts these residuals.

**Figure 7.**
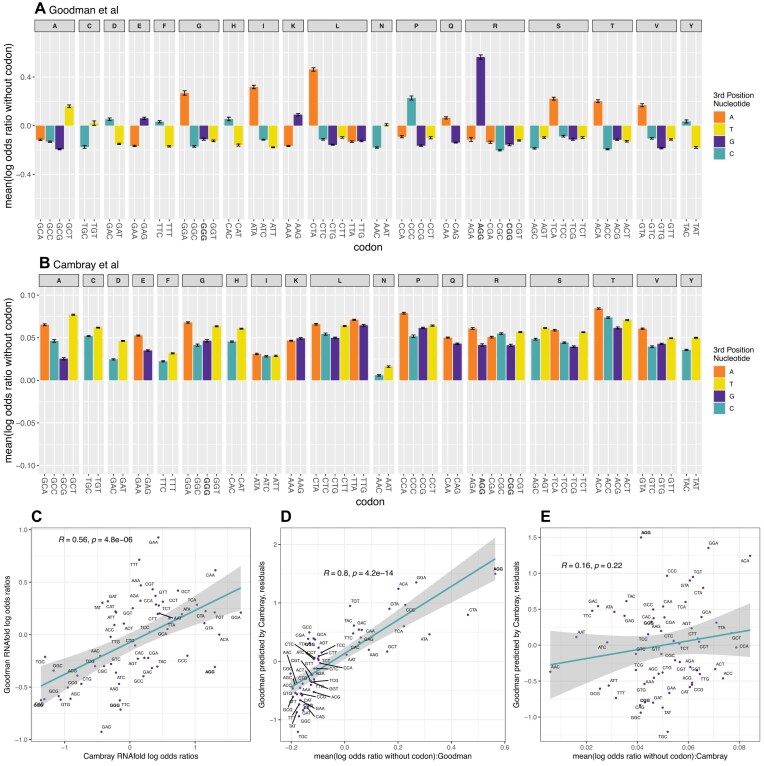
The relationship between non-random codon allocation to constructs and deviation between datasets using RNA stability log odds ratios. RNA stability measures refer to computational predictions using the Vienna RNA R package [44]. For the Goodman (**A**) and Cambray (**B**) construct sets we considered, for each construct containing a given codon, the mean log odds ratios of the other codons in the same construct. We calculate the mean of these values for each construct and then the mean of means for all constructs containing the focal codon. As can be seen, the Goodman data have some highly deviant data points, notably AGG whose flanking codons have a highly positive log odds ratio (i.e. feature in the most unstable RNA). To consider whether these deviations explain the differences between Cambray and Goodman log odds ratio, we consider the regression between the two (**C**), this time deriving the regression line of the Goodman data predicted by the Cambray data. We then determine whether the residuals from this regression are predicted by the mean log odds of the flanking codons in the Goodman data (**D**) and the Cambray data (**E**). Statistics in C–E are Spearman rank correlations. Error bars in panels A and B are standard error of the mean (SEM). NGG codons are highlighted in bold.

As before when considering protein level metric, we find that the Goodman passenger effects predict the residuals (Fig. 7D) but the Cambray passenger effects do not (Fig. 7E). We find the Goodman passenger effects to explain between 71% (Spearman correlation = 0.84, Fig. 7D) and 64% (Pearson correlation = 0.80) of the difference between the two datasets, whilst the Cambray explains between 2% (Spearman correlation = 0.14, Fig. 7E) and 3% (Pearson correlation = 0.16), and neither of the two Cambray correlations is significant. In the Goodman data, AGG is differentially found in constructs whose codons are enriched in low stability 5′ ends (Fig. 7A), despite the fact that it should *a priori* be associated with high stability.

### No evidence that 5′ non-optimal codon usage promotes protein levels

Our original ‘biological’ hypothesis to explain why AGG may be an outlier [34] is related to the influential conjecture [35] that non-optimal codon usage at the 5′ end slows ribosomes and that this, in addition to RNA stability effects, boosts protein level (see also [48]). That AGG is the most non-optimal of all codons in *E.coli*, it being greatly enriched in the core of *E. coli*’s native lowly expressed genes (nLEGs) compared to nHEGs, renders such an hypothesis attractive. While the exceptional status of AGG in the Goodman data appears to be in large part an artefact, this doesn’t exclude the more general possibility that codon rarity is influential in determining transgene efficiency. More generally, what features of the early CDS are relevant? To control for covariances, one approach is to consider partial correlations [20, 39]. This applied to ribosomal density data [20] suggests that at 5′ ends RNA stability, positive charge and codon adaptation all contribute to promoting orderly ribosomal initiation, with non-optimal codons argued to slow ribosomes to prevent collisions, so enabling higher fitness and potentially higher protein output per RNA.

To further address this question, we consider, for Cambray’s dataset, features that may be predictors of protein per RNA levels. A common practice has been codon optimization of transgenes in which metrics like the CAI are employed to inform transgene design [33, 49–51]. Whilst the original ramp hypothesis [35] has commonly been interpreted as predicting 5′ codon adaptation in the first 10 codons to be influential on expression [2, 5, 14, 21], it has also been suggested that it is not necessarily true in absolute terms, but rather that the differences in codon adaptation as one moves further into the gene body is what matters [39]. We thus also consider delta CAI i.e. the difference in CAI between the first 10 codons following the start codon and the 20 codons downstream (the full manipulated sequence provided in Cambray data). On top of the codon adaptation degree, we consider RNA structure, the proportion of NGG codons in the first 6 codons, proline content and density of positively charged amino acids in the first 10 codons, all having been considered as potential modifiers of ribosomal velocity or to affect initiation (see e.g. [4, 10, 20, 27–29, 38, 52–54]). We assume lysine, arginine and histidine are positively charged [55]. In addition, we include GC content of the first 30bp to allow for unaccounted stability effects (as do Cambray *et al.* [7]). To determine the codon adaptation degree (for our CAI and delta CAI) we consider, as with the classical CAI, the enrichment of codons in nHEGs. Unlike the classical CAI, we compute a log odds ratio for all codons from the 10 codons at genic cores and employ amassed protein abundance data. The two codon adaptation metrics correlate well (see [34]).

In a linear model, all variables are highly significant (there are 232 833 degrees of freedom so even miniscule effects are highly significant) and explain 21.39% of the variation in protein per RNA. Relaimpo analysis [45] allows us to partition relative importance of the predictors in terms of the proportion of the explained variance, explained by each of the predictors allowing for covariance. This reveals an order of stability (-30bp to + 30bp) explaining 72.5%, GC at 15.8%, stability for 0bp to 60bp at 6.9%, then proline content at 3.98%. All other variables explain less than 1% of the explained variance: positive charge (0.33%), CAI (0.24%), delta CAI (0.19%), and NGG content (0.16%). In this model (using Z score normalised values), higher CAI levels are predictive of more protein per RNA, albeit weakly so.

As an alternative method, we consider partial correlation analysis, which has been previously used in similar contexts [20]. After control for other variation, codon adaptation is again significantly positively correlated (Table 1 reports for Spearman’s correlation, and also seen using Pearson partial correlations). We thus find that, rather than slowing ribosomes by employment of non-optimal codons, if anything, greater usage of the translationally optimal codons predicts very slightly higher transgene output. Thus, it is unlikely that any part of AGG’s association with well-expressed transgenes in the Goodman data was owing to ribosomal slowing effects mediated by low codon adaptation. However, as AGG also codes for arginine, a positive charge effect may yet contribute to a minute degree. The delta CAI trend follows the predicted direction both before and after covariate control, where a lower 5′ CAI compared to downstream is associated with higher protein per RNA. However, the effect size is even smaller than that of absolute CAI measures (Table 1). As Cambray *et al.* also provide measures of cell growth rates, we also further investigate the effect of potential ramp predictors on cell fitness and find that on this too they have minimal effects (Supplementary Table S4). Cambray *et al.* present similar observations, reporting that CAI and codon ramp bottleneck position and strength had very small effects on protein production and growth in their study [7].

**Table 1.** Spearman correlations and partial correlations of potential predictors on protein per RNA in Cambray data. Protein per RNA refers to production with normal translation initiation (PNI/RNA_SS_). Stab30 is the stability of the construct from −30 to +30, likewise stab60 for 0 to +60 bp (both metrics reported by Cambray *et al.*), CAI is the log odds ratio for each codon reflecting codon enrichment in the core of highly expressed native genes, assayed using protein abundance (see [34]), deltaCAI is the difference between the CAI metric for 5′ ends (taken to be the first 10 codons following the start codon) and that of the rest of the CDS, NGG is the density of NGG codons in the first 6 codons (where N can be any of the four nucleotides), Pro is proline density in the first 10 codons, Pos is the density of positively charged amino acids, and GC is the G + C nucleotide content of the first 10 codons. Significance (*P*-value) is displayed with a ranging number of stars so that: *P* < .0001 = “****”“, *P* < .001 = ”*** “, *P* < .01 = ”** “, *P* < .05 = ”* ", *P* > .05 = no stars. The upper quadrant along the table diagonal is for correlations, the lower for the partial correlations. The first column is the prediction of protein per RNA allowing for covariation

	Prot_per_RNA	stab30	stab60	CAI	deltaCAI	NGG	Pro	Pos	GC
Prot_per_RNA	−	0.42****	0.14****	−0.01****	−0.02****	−0.02****	−0.09****	−0.02****	−0.21****
stab30	0.42****	−	0.04****	−0.02****	−0.02****	−0.02****	0.02****	0.01***	−0.08****
stab60	0.11****	−0.02****	−	0.01**	0.01****	−0.05****	0.01****	0.01**	−0.17****
CAI	0.03****	0	0.02****	−	0.73****	−0.12****	−0.04****	−0.05****	0.23****
deltaCAI	−0.02****	0.00*	0.02****	0.71****	−	−0.07****	−0.06****	−0.07****	0.17****
NGG	−0.01**	0	−0.03****	−0.12****	0.02****	−	−0.13****	0.05****	0.03****
Pro	−0.07****	0.07****	0.08****	−0.07****	−0.06****	−0.15****	−	−0.21****	0.32****
Pos	−0.04****	0.03****	0.03****	−0.01****	−0.06****	0.01*	−0.23****	−	−0.01**
GC	−0.14****	−0.01****	−0.17****	0.18****	0.02****	0.10****	0.35****	0.08****	–

Cambray *et al.* also provide a measure that reports protein levels accounting for 5′ structural effects (termed “PFI”), which could be of use in identifying the influence of additional predictors of expression. We thus repeat the relaimpo [45] (Supplementary Fig. S8) and partial correlation analysis (Supplementary Table S5) employing PFI by RNA instead of PNI by RNA, and report similar results as above. Unlike previously, we find stability 0 to +60bp to be by far the strongest predictor on PFI by RNA (explaining over 80% of the effect), whilst the effect of stability −30 to 30 bp is diminished (Supplementary Fig. S8). As the PFI measure controls for potential secondary structures within the initiation region, it is expected that we are now detecting stability effects further along the CDS. Nonetheless, we find that the direction of the correlations between protein by RNA and all other predictors remains unchanged. Codon adaptation within the first 10 codons is significantly weakly positively correlated to protein by RNA even when the protein measure inherently controls for 5′ structural effects (Supplementary Table S5), again suggesting that use of translationally non-optimal codons likely does not predict increased transgene output.

### Native HEG 5′ codons are not enriched in the Cambray transgenes

Given that the Goodman data provide biased estimates for codon-protein production trends, might prior results be unsafe? In particular, it has been reported that the codons enriched at the 5′ ends of the most effective transgenes in the Goodman data were avoided in nHEGs compared to nLEGs [34]. Is this result too an artefact? Is then the same trend seen in the Cambray data? As the Goodman data employed up to codon 11, we repeat the same. Codon 11 is also approaching the limit at which the 5′ end has influence on protein levels (in relaimpo analysis [45] GC content at codon 11 is greater than null in its influence of protein level in Cambray data (see Supplementary Fig. S4, [3]).

One issue is the correct metric to employ for the native genes’ expression. For consistency, we also consider native protein per RNA (where protein abundance data are obtained from PaxDB [42], and RNA levels are derived from a compendium of RNA-seq data for *E. coli* [43]), but consider RNA metrics in four different ways: using either mean of all measures with “no data” treated as zero, mean of all measures with “no data” treated as NA, median of all measures with “no data” treated as zero, or median of all measures with “no data” treated as NA. As expected, the four metrics produce a highly positively correlated set of native codon enrichment trends (Supplementary Fig. S9).

First, we replicate the prior result that employed protein level and Goodman's Prot.FCC but now using the above native protein/RNA metrics and, in addition, Goodman's Trans data, giving 10 comparisons. All show the same negative correlation, as originally claimed, i.e. codons enriched in tHEGs at their 5′ ends are avoided in nHEGs (Supplementary Fig. S10).

**Figure 8.**
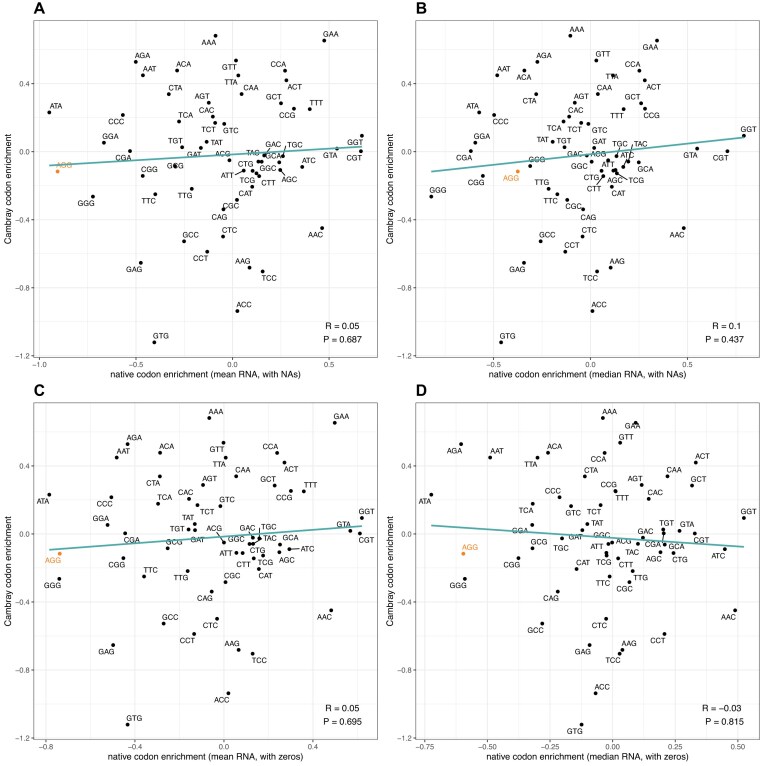
Comparison of codon enrichment log odds ratios between Cambray protein/RNA and native genes. For Cambray, protein/RNA measures are considered in each plot (*y*-axis). Different metrics are used for native protein/RNA measures, where native RNA data is considered in four ways: (**A**) mean of all measures with no data treated as NA, (**B**) median of all measures with no data treated as NA; (**C**) mean of all measures with no data treated as zero; (**D**) median of all measures with no data treated as zero. Plots display a linear regression and Spearman correlation with respective *P*-value. All native protein data was obtained from PaxDB [42] and cross-referenced with RNA measures from a compendium of RNA-seq datasets [43].

Next, we ask whether this lack of concordance is seen when employing the Cambray protein per RNA metric. We find that comparing the enrichment of each codon in the 5′ domain of nHEGs against the enrichment seen in the Cambray data, there can be either a very weak positive or weak negative correlation, none even close to significance (Fig. 8). While it does not replicate the significant anti-correlation seen for Goodman (Supplementary Fig. S10, [34]), *prima facie* this supports the hypothesis that codons enriched 5′ in nHEGs compared to nLEGs are not the same codons enriched 5′ in transgenes, comparing the most highly expressed (protein per RNA) to the least expressed.

### Cambray log odds ratios well predict codon usage across 5′ ends, compared to gene cores, for non-AT rich bacteria

In addition to the above, it was previously observed that, based on the Goodman data, 5′ ends of genes of *E. coli* and some other bacteria are enriched, compared to gene cores, for codons enriched in the most tHEGs [34]. This is a potentially important insight for transgene design in non-model organisms suggesting that the transgene data may have application. It also agrees with the thesis that codon usage at 5′ ends is dominated by selection for low stability (which should be universally associated with AT richness) rather than any specific of codon-tRNA usage patterns. However, since we have now established that the Goodman data is distorted by passenger effects, is the prior result robust?

**Figure 9.**
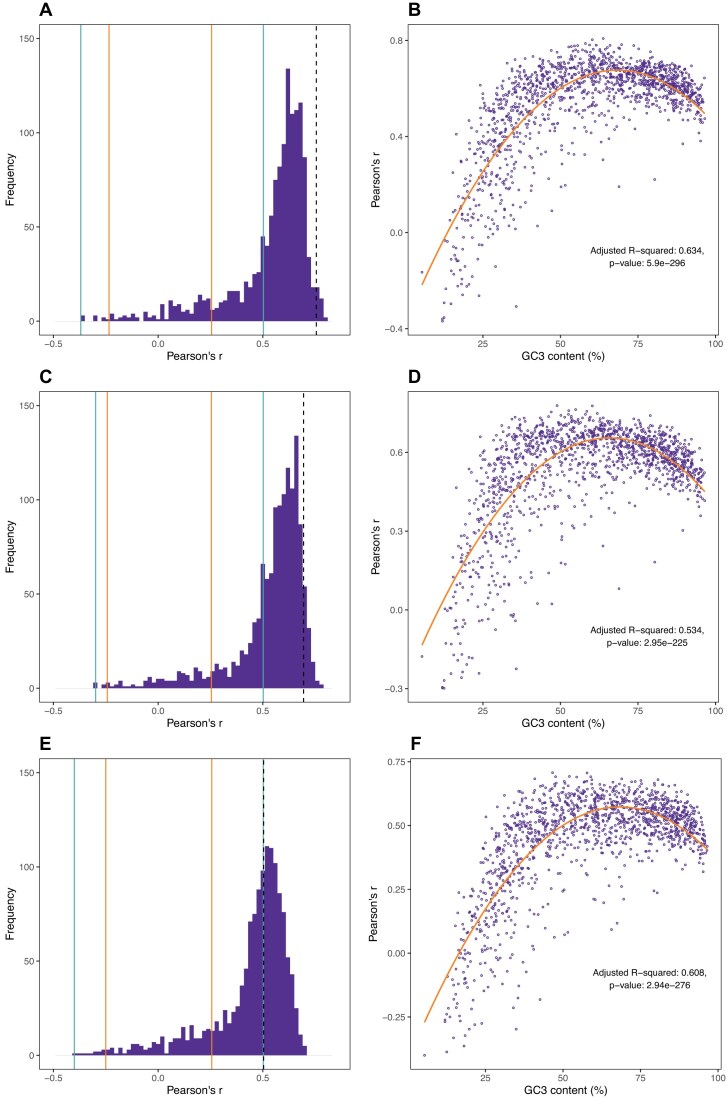
Comparison between 5′ v core codon enrichment and tHEG codon enrichment in 1355 bacterial genomes. (**A**) Histogram of Pearson correlations between the log odds ratios of 5′ v core codon enrichment of the bacterial genomes and the codon enrichment in highly v lowly expressed transgenes in the Goodman data (by protein measure, Prot.FCC). Displayed are vertical solid lines to show statistically significant correlations (inner orange lines for *P*< 0.05 and outer light blue lines for significance after Bonferroni correction). Vertical black dashed line represents *E. coli*. (**B**) The same Pearson correlations predicted by mean GC3 of all genes in a genome. A quadratic model best fits the prediction, its adjusted R^2^ and *P*-value are displayed on the figure. Panels (**C**) and (**D**) are the same as A and B but for Goodman’s protein/RNA measure (Trans). Panels (**E**) and (**F**). are the same as A and B but for Cambray rather than Goodman-derived log odds ratios (protein/RNA).

To determine the robustness, we consider for both Cambray and Goodman the log odds ratio vector of enrichment in the most tHEGs (as in Fig. 2). For each 59 element vector, we then compare it to a 5′ v core log odds ratio enrichment vector for each of 1355 bacterial genomes (Supplementary Table S3), this being an expansion on the previously employed 650 [34]. For both datasets, we see a large number of genomes whose 5′ enrichment trend correlates well with both the Goodman (with Trans and Prot.FCC) and the Cambray vectors (Fig. 9). However, for the Cambray data, there are many bacterial genomes with higher correlations than seen for *E. coli*, the same not being true for the Goodman data (Fig. 9). For both, a quadratic fit of correlation coefficient against GC3 robustly predicts the data. For the Cambray data, only really the most AT rich genomes do not show a robust correlation (Fig. 9F). This likely reflects the fact that selection for high AT content at the 5′ end is indistinguishable from background AT content pressures in such genomes. We conclude that prior results concerning prediction of 5′ v core enrichment trends across bacteria [34] are not importantly affected by passenger effects.

## Discussion

As Bateson recommended [1], we should treasure our exceptions. In this context, AGG’s exceptionally strong association with high protein output in the Goodman *et al.* data, when prior data suggested that it should be suppressive, demands explanation. Our results suggest a simple explanation, namely that AGG’s high score in the Goodman data is an artefact of transgene non-random codon construction. Commensurate with this, we find limited agreement between the datasets, agreement that becomes ever weaker as the level of analysis becomes ever more focused. At the point where indeed we ask which codon for any given amino acid is preferred at any given position, the agreement is barely more than expected by chance. In part, this may reflect sample size issues in the Goodman data. Indeed, for some amino acids at some positions, a score cannot be given owing to the absence of data. Nonetheless, even when considering trends seen across the full 11 first codons (start codon + 10), there is considerable noise and some profound disagreement, most notably for AGG, despite the fact that, in principle, the two studies are examining the same issue in the same species using very similar approaches.

We show that the Goodman constructs are highly non-random in design, with AGG being an outlier (Fig. 5). We estimate that passenger effects explain up to 70% of the disagreement between the two datasets (Fig. 7D), so the unexplained discrepancy accounts for just 30%. In retrospect, these passenger effects are to be expected as Goodman *et al.* designed some constructs to sit at extremes as regards to stability and codon usage, such design by necessity introducing non-random associations between codons and hence forcing passenger effects. Similarly, Cambray *et al.* note that Kudla et al.’s constructs [4] introduced some rare codons in their design, which led to the mistaken conclusion that codon rarity was causative of low expression [7].

While then the Goodman *et al.* data provides robust guides to general 5′ trends promoting protein expression, their codon-by-codon data is problematic. This underscores the notion that the size of the dataset is not alone a panacea for the unbiased design of transgenes. How might one mitigate passenger effects is then also a relevant question. As we note, part of the issue may well be that they designed constructs to adopt certain profiles, which is likely to be enabling of passenger effects. Full randomization is thus preferable. More particularly, we suggest that our focal codon exclusion tests provide a basis for a diagnostic of passenger effects.

What remains uncertain is the extent to which the Cambray data is also affected by passenger effects. It is striking that so many codons in the Cambray constructs have positive passenger effect scores (Fig. 6B). Likewise, codons are not randomly associated within constructs (Fig. 5A), though to a much lesser degree than in the Goodman data (e.g. Fig. 5C). One hypothesis for this is that a truly randomized construct library will contain constructs that never experimentally express. If these cannot be scored, or are otherwise eliminated from analysis, then the extant set of constructs will be biased towards those that have some level of expression. This eliminates a set of constructs in which, for many non-focal codons, the construct is composed mostly of low protein production codons (non-zero though still low protein titre, meaning negative log odds score), so upwardly distorting the mean of the log odds ratio. If true, then the null expectation isn’t a mean of zero. However, Cambray *et al.* present (and we analyse) the sequences of all 244 000 constructs. While 11 159 have not enough data with which to determine protein/RNA, unless some constructs were never considered as they were likely to not express, the above problem does not provide an explanation. We also consider the possibility that the lack of random codon association is due to the fact that several of Cambray’s constructs contain the same sequence at codons 2–11. However, limiting our focal codon exclusion test to the 125 067 different 5′ CDSs (with again 1000 random selections of 14 234 constructs to match Goodman's sample size), our observation that codons are not randomly associated within constructs persists (Supplementary Fig. S11), meaning this too does not provide an explanation. As expected, native genes tend to employ 5′ codons that are non-randomly flanked by codons that promote protein production (Supplementary Fig. S12).

### Further evidence for negative AGG effects and resilience to experimental variation

While we find that construct non-random design in the Goodman data explains a good proportion of discrepancy in log odds ratio values associated with each codon (up to ∼70%), we have made no attempt to explain the remaining variance. Some will be stochastic variation and measurement error. It seems reasonable to also expect that the differences in log odds ratios between the two datasets are at least partially owing to differences in experimental approaches, including differences in growth conditions, expression systems, strains employed etc. For example, Cambray *et al.* used IPTG-inducible expression in *E. coli* MDS42 [7], while Goodman *et al.* used constitutive expression from plasmid in *E. coli* MG1655 [2]). Equally, Cambray *et al.* relied on polysome profiling and RNA sequencing to directly assess translation efficiency [7], whereas Goodman *et al.* used FlowSeq [2]).

Variation between constructs that differ in amino acid content (as with both Cambray and Goodman) may also be condition sensitive as, for example, Osterman *et al.* have shown that codons for costly amino acids are more influential under nutrient deprived conditions [14]. The Osterman *et al.* experiment [14] also permits us to ask about the extent to which environmental conditions will affect log odds ratios ascribed to any codon. These authors vary amino acid and codon usage in 10 codon blocks placed between the start codon and a reporter construct [14]. From this they derive protein level, compared to a control reporter, for each construct (for details see 'Materials and methods'). Unfortunately, they don’t provide RNA level data and the protein levels are presented on a rough 1–5 integer scale, making quantitative comparison with the other two datasets problematic. While not then optimal for comparison with the Cambray data, as they run an otherwise identical experiment in LB and M9 minimal media, we estimate the effect that radically changing the growth media has on synonymous codon-centred log odds ratios. We can also ask whether AGG is an outlier in this dataset too.

We find that log odds estimated under the different growth conditions are highly similar (Pearson correlation = 0.95, Supplementary Fig. S13), suggesting striking media insensitivity. Indeed, if we bootstrap sample the high and low expression constructs from the normal media samples (LB), we find that the 95% limits on correlation between estimated log odds ratios of these bootstraps is lower than the correlation observed between the two different media (Supplementary Fig. S14). This suggests that the differences between media are no greater than those expected from stochastic variation within media. While the original authors identify major amino acid level affects [14], this additionally underscores the utility of comparing within synonymous blocks in our log odds ratio approach as it mitigates any amino acid dependent effects, including those arising from differences in experimental set-up.

In both the normal and minimal media datasets, AGG and the other NGG codons, GGG and CGG, score negatively (Supplementary Fig. S13), as they do in the Cambray data. This further underscores the unusual nature of the Goodman *et al.* result.

Further analysis of the role of experimental set-up may be valuable not least because these large-scale transgene studies not only provide broadscale trends (on which they largely agree, with RNA stability being key), but can also be employed to specify which codon to best employ at any given position in CDS of a transgene [14]. That the Cambray and Goodman studies show so little agreement (marginally more than chance (Fig. 4)) is then a concern.

### The (enigmatic) effect of rare codons on transgene efficiency

Since the first bacterial sequences became available, it was recognised that *E. coli* employs otherwise rarer codons, those that are non-optimal as regards translation, at their 5′ ends [15]. A low rate of evolution at synonymous sites indicated that selection was likely to be operating beyond the level of the protein [15]. The meaning of non-optimal codon usage, however, has been in dispute, the central tussle being between those who consider rarity as being functionally important as rarer codons slow ribosomes [35], versus those who consider the over-representation of rare codons in the 5′ ends of native transcripts and in tHEGs as being a consequence of effects promoting low RNA stability (A enrichment), any effects on codon rarity being functionally irrelevant. What is unambiguous, and shown repeatedly [see e.g. (2, 4, 5, 7, 8, 14, 21, 53, 56–58)], is that low 5′ stability of the mRNA is overwhelmingly the major predictor. Goodman *et al.* [2] find that, controlling for stability, codon bias does not predict protein level, but as we have shown there may be an issue with construct design. Bentele *et al.* [21] make comparable claims showing that the rare codon enrichment is particular to those that would also cause low stability. Osterman *et al.* [14] report no effect of tRNA availability on transgene productivity [see also (59)].

In this context, we assumed that our multivariate approach would decide between whether highly efficient transgenes are predicted by low codon adaptation (the functionally relevant model) or not dependent on codon adaptation (the incidental byproduct model). We didn’t expect to see that two methods (regression with relaimpo analysis and partial Spearman correlation analysis) would both report the same trend, namely that higher codon optimality in the 5′ end predicts higher protein per RNA (Table 1). Even in Cambray’s constructs that attempt to remove the influence of 5′ RNA stability, we see a positive correlation (Supplementary Table S5). However, all such correlations are so weak that the most that we would like to conclude is that, after covariate control, there is no support for the hypothesis that rarer codons promote protein productivity.

Where does this leave the ramp hypothesis? In its initial incarnation, it implicated a role for absolutely rare codons [35], an interpretation taken up since [see e.g. (2, 5, 14, 21)]. However, an alternative interpretation emphasises that the rarity of codons need merely be lower in an initial 5′ domain than in the following sequence [39]. In principle, high adaptation of the initial 5′ domain could yet be consistent if the highest transgene output is associated with even higher codon adaptation in the downstream region. To consider this, our model also includes delta CAI i.e. the difference in codon adaptation scores 5′ and in the codons following in the Cambray data (i.e. CAI in the first 10 codons following the start codon v CAI in the 20 codons downstream) [7]. Without the addition of delta CAI, the linear model has an adjusted R^2^ of 0.2136, with it, the adjusted R^2^ is almost identical at 0.2139. While the trend is in the predicted direction (before and after covariate control) with a lower 5′ CAI than downstream predictive of higher protein per RNA, the effect is extremely small (Spearman rank partial rho squared = 0.0004, Table 1). In addressing whether putative ramp effects mediate cell fitness, which is associated with ribosomal hogging and wastage, we also see very minimal effects on cell growth (Supplementary Table S4). More generally, most predictors considered part of the ramp (positive charge, CAI, delta CAI) are so small in effect size (Table 1, Supplementary Table S4) that their biological relevance must be questioned. Our results come with the caveat that, due to sequence availability within the Cambray data [7], we consider feature differences within the first 30 codons following the start codon, whilst larger effects could be captured if considering sequences further downstream (e.g. it has been suggested to be an effect present through to codon 50 [35, 39]). Though, our results reify the consensus [2, 5, 7, 14, 21] that the ramp hypothesis appears to be largely irrelevant to transgene design if protein production is the focal concern.

What of other possible determinants of protein level? As regards Shine-Dalgarno mimics, Osterman *et al.* [14] suggest that, along with AGG, codons GGG and GGA also have strong binding potential to the Shine–Dalgarno antisense RNA and thus have the potential to contribute strongly to Shine–Dalgarno mimic sequences. If these have negative scores, then, Osterman *et al.* propose, this would be consistent with suppressing effects of Shine-Dalgarno mimics [14]. However, GGA has a positive log odds ratio in the Cambray data (Fig. 2B), only GGT in the glycine block having a higher score, this also being a potential mimic, Shine-Dalgarno terminating GGU. More generally, the codons that feature highest as potential Shine-Dalgarno mimics are all G rich, and G richness is associated with high stability, thus presenting a further case where covariates obscure interpretation. Additionally, the strong Shine-Dalgarno mimic GGG is typically avoided [60], possibly owing to G quadruplex initiation [61]. Further covariate controlled analysis is needed to robustly test the Shine-Dalgarno mimic hypothesis that we leave to future work.

### Why do native HEGs not employ those codons associated with high transgene expression?

There is a common, hard to disavow [62], presumption that codon enrichment trends in nHEGs reflect trends that are causative of high expression and thus should be designed into transgenes. However, transgene evidence was argued not to be reflective of native genes as they represent more extreme RNA stability than seen natively [39, 40]. Linked to this, that translation initiation in native genes was shown to only explain 1% of translation efficiency [63] further supports the difference with transgenes (in which 5′ ends were shown to have a disproportionate effect on protein production, with reduced mRNA stability as a proposed mechanism [2–5, 7, 8, 11–14]). It has also been known for a considerable time that, while nHEGs over-employ in their gene body (not 5′ ends) those codons matching the more abundant tRNAs [21], enrichment of transgenes with such translationally “optimal” codons usually has little to no effect on protein level [2–4, 7, 9]. The selection on native genes to incorporate “optimal” codons is more likely to be selection to release ribosomes faster, hence permit processing of other transcripts, thence affecting fitness [4, 6, 7]. As it has been shown that nHEGs show overall higher codon optimality than nLEGs [39] (though the 5′ v core optimality difference is present regardless of gene expression levels [39]), there could be an explanation for our results demonstrating that higher codon optimality is weakly associated to higher transgene expression. Though, more recently, while the importance of 5′ ends has been long recognised [12, 13], enrichment trends in nHEGs at 5′ ends have also been shown [34] to not be concordant with enrichment trends seen in one set of transgenes designed to consider exclusively these 5′ effects [2]. Here we sought also to ask whether other transgene datasets support the same conclusion. We find that the second dataset [7] reinforces the prior claim [34] that codons enriched 5′ in nHEGs are not concordant with those enriched in tHEGs (Fig. 8).

This latter result underscores the notion that selection on codon usage in nHEGs need not solely be focused on maximization of protein level per RNA. Importantly, other factors, such as noise reduction and ribosomal efficiency, are both important and potentially at odds with maximization of ribosomal initiation rates [34, 63, 64]. Guimaraes *et al.* [63], in analysing native *E. coli* data from Taniguchi *et al.* [65], find that lower mRNA levels and higher translational efficiency are predictive of higher noise. Low noise was theorised to be the result of low ribosomal density [66], and indeed higher numbers of ribosomes per transcript are predictive of increased abundance-corrected noise [67] (but see [68]). This may explain why many important bacterial genes have conserved inefficient ribosomal binding sites [67]. Selection for optimal usage of the available ribosomes can also select for low ribosomal density on highly expressed transcripts [69], as similarly predicted by the noise model. For both reasons, we need not expect that nHEGs and tHEGs would necessarily agree on which codons need be the best in 5′ domains, again underscoring the notion that nHEGs are not simply under selection for protein titre maximization.

The effect of codon usage is also not limited to the translation stage, and can be under selection to favour other aspects of gene expression (discussed thoroughly in the literature, see e.g. [34, 70–72]). Moreover, cellular fitness, not simply protein production efficiency, may be important. Indeed, avoidance of catastrophic random declines in abundance of essential proteins may well be of relevance for native genes. For the production of transgenes, the stochastic effects are probably irrelevant as the products are not essential for cellular functioning, but consideration of cellular costs may be relevant.

## Supplementary Material

lqaf086_Supplemental_Files

## Data Availability

All input data and downstream scripts, both processing and figure/statistical analyses are available at https://doi.org/10.5281/zenodo.15084715. Supplementary Data is available at NAR Genomics & Bioinformatics online.
