# Supplementary material for: Why AGG is associated with high transgene output: passenger effects and their implications for transgene design
Source: NAR Genom Bioinform. 2025 Jun 19;7(2):lqaf086. doi: 10.1093/nargab/lqaf086 (PMC12204400; doi:10.1093/nargab/lqaf086)
Supplement: lqaf086_Supplemental_Files [file lqaf086_supplemental_files.zip › Supplementary_figures_corrected.pdf]

Supplementary figures

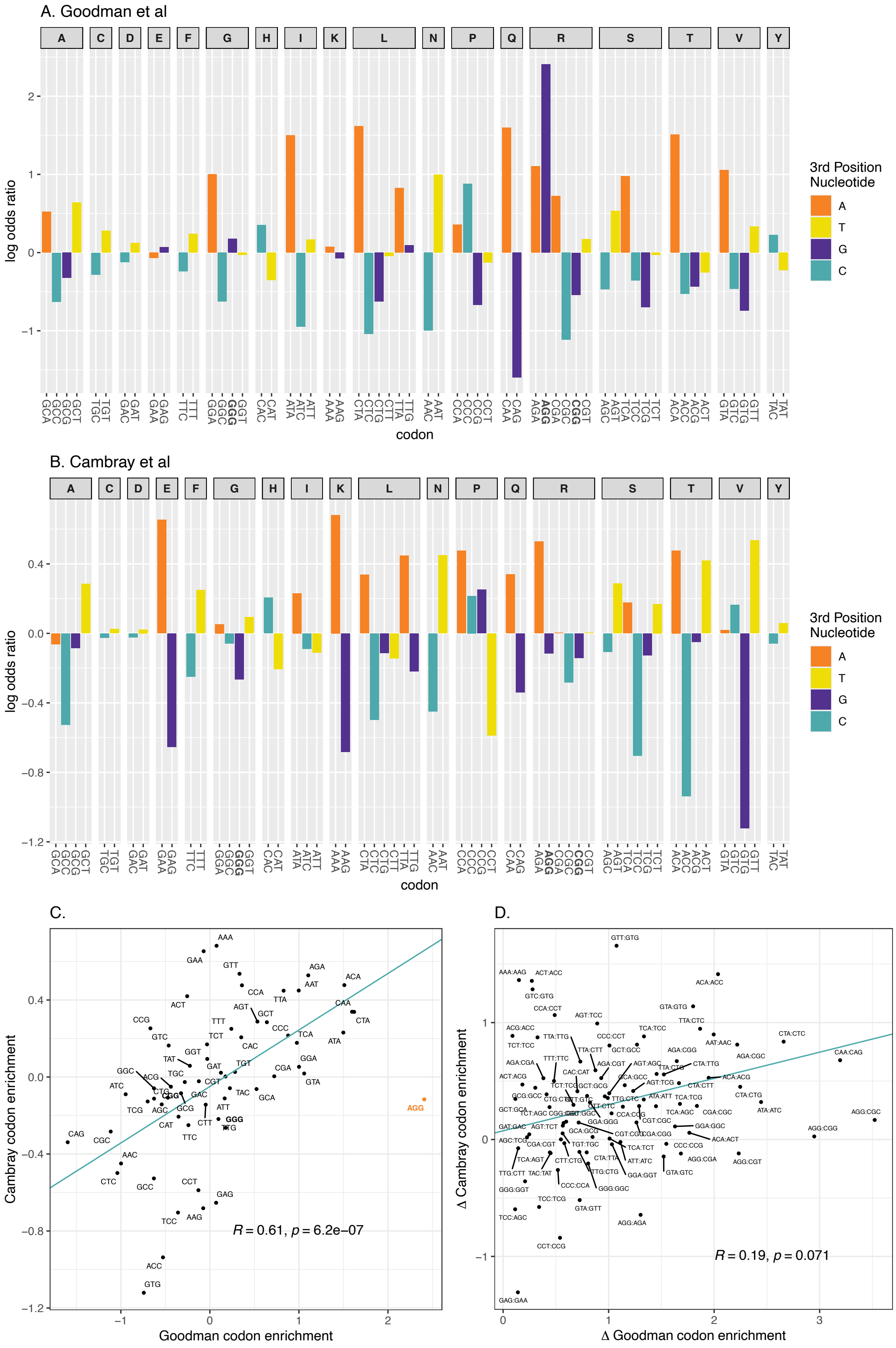

Fig S1. Cambray v Goodman overall 5' codon preferences in highly v lowly expressed transgenes (all 10 positions following the start codon). Log odds ratios for enrichment in the top quartile by expression for each codon within a synonymous codon block for **A.** Goodman data (using Prot.FCC) and **B.** Cambray data (protein per RNA). **C.** The x axis is the log odds ratio for the codon being enriched at the 5' ends in Goodman transgenes with high protein per RNA compared to low protein per RNA, the y axis is for Cambray transgenes. Each data point is labelled as the codon it represents, AGG is marked in orange. For panels A, B and C, NGG codons are highlighted in bold. **D.** As for panel C, but comparing all pairwise combinations of synonymous codons (i.e. within the same codon block: N=87). The pairwise differences are oriented such that, on the x axis, the codon with the lower value of the log odds ratio has its value subtracted from that of the higher value. The orientation is preserved for the y axis. Each point is labelled by the oriented codon pair (first codon in the pair has the higher x axis value, as seen in panel C). For both C and D, Principal Components Analysis (PCA) was used to fit an orthogonal regression line, and the Spearman's correlation coefficient and p-value are provided within the plots.

Goodman

Amino Acid / Position	2	3	4	5	6	7	8	9	10	11
Y	NA	TAC	TAT	TAC	TAT	TAC	TAT	TAT	TAC	TAT
V	GTT	GTA	GTA	GTA	GTT	GTA	GTA	GTT	GTA	GTA
T	ACA	ACA	ACA	ACA	ACA	ACA	ACA	ACA	ACA	ACA
S	AGT	TCA	TCA	TCT	TCA	TCA	AGC	AGC	TCA	TCA
R	AGA	AGA	AGA	AGA	AGA	AGG	CGT	AGG	AGG	AGG
Q	CAA	CAA	CAA	CAA	CAA	CAA	CAA	CAA	CAG	CAA
P	CCT	CCT	CCC	CCA	CCT	CCC	CCC	CCA	CCA	CCC
N	AAT	AAC	AAT	AAT	AAT	AAT	AAT	AAT	AAC	AAT
L	CTA	TTA	TTA	TTA	TTA	TTA	CTA	CTT	CTG	CTA
K	AAA	AAA	AAA	AAA	AAA	AAA	AAA	AAG	AAA	AAG
I	ATA	ATA	ATA	ATA	ATA	ATA	ATA	ATA	ATA	ATA
H	CAC	CAT	CAC	CAC	NA	CAC	CAC	NA	CAT	NA
G	GGA	GGG	GGA	GGG	GGT	GGA	GGA	GGA	GGA	GGG
F	TTC	TTT	TTT	TTT	TTT	TTC	TTC	TTT	TTC	TTC
E	GAA	GAA	GAA	GAA	GAG	GAA	GAG	GAG	GAG	GAA
D	GAC	GAT	GAT	GAC	GAT	GAT	GAT	GAT	GAT	GAC
C	NA	TGC	NA	NA	TGC	TGC	TGT	TGC	TGT	NA
A	GCA	GCT	GCA	GCA	GCT	GCG	GCA	GCT	GCC	GCA

Cambray

Amino Acid / Position	2	3	4	5	6	7	8	9	10	11
Y	TAT	TAT	TAT	TAC	TAC	TAT	TAC	TAC	TAC	TAC
V	GTT	GTT	GTT	GTT	GTT	GTT	GTT	GTT	GTA	GTT
T	ACT	ACT	ACA	ACA	ACA	ACA	ACA	ACT	ACT	ACT
S	AGT	AGT	TCA	TCA	AGT	AGT	AGC	TCA	AGC	TCG
R	AGA	AGA	CGA	AGA	AGA	AGG	AGA	AGA	AGA	CGG
Q	CAA	CAA	CAA	CAA	CAA	CAG	CAA	CAA	CAA	CAG
P	CCA	CCA	CCA	CCA	CCG	CCC	CCA	CCA	CCC	CCG
N	AAT	AAT	AAT	AAT	AAC	AAT	AAT	AAC	AAT	AAT
L	TTA	TTA	TTA	TTA	CTC	TTA	CTA	TTA	TTG	CTG
K	AAA	AAA	AAA	AAA	AAA	AAA	AAA	AAA	AAA	AAA
I	ATC	ATT	ATA	ATA	ATT	ATA	ATA	ATA	ATT	ATC
H	CAT	CAC	CAC	CAC	CAC	CAC	CAT	CAC	CAC	CAC
G	GGG	GGC	GGT	GGT	GGC	GGA	GGA	GGA	GGT	GGA
F	TTT	TTC	TTT	TTC	TTC	TTT	TTT	TTT	TTT	TTC
E	GAG	GAA	GAA	GAA	GAG	GAA	GAA	GAA	GAA	GAA
D	GAT	GAT	GAT	GAC	GAC	GAT	GAT	GAC	GAC	GAC
C	TGT	TGT	TGT	TGC	TGC	TGT	TGC	TGC	TGC	TGT
A	GCT	GCG	GCT	GCC	GCT	GCC	GCT	GCG	GCT	GCT

Fig S2. Cambray and Goodman most enriched codon, for each amino acid, in highly v lowly expressed transgenes, broken down by 5' position. Transgene expression refers to protein/RNA metrics in both datasets. Position refers to codon position after the start codon.

Goodman

Amino Acid	2	3	4	5	6	7	8	9	10	11
Y	TAC	TAC	TAT	TAC	TAC	TAC	TAT	TAT	TAC	TAC
V	GTT	GTA	GTA	GTA	GTT	GTA	GTA	GTA	GTA	GTA
T	ACA	ACA	ACA	ACA	ACA	ACA	ACA	ACA	ACA	ACA
S	AGT	TCA	TCA	TCA	TCA	TCA	TCC	AGC	TCA	TCA
R	AGA	AGA	AGG	AGG	AGA	AGG	AGG	AGG	AGG	AGG
Q	CAA	CAA	CAA	CAA	CAA	CAA	CAA	CAA	CAA	CAA
P	CCA	CCA	CCC	CCA	CCC	CCC	CCC	CCG	CCC	CCC
N	AAT	AAT	AAT	AAT	AAT	AAT	AAT	AAT	AAC	AAT
L	CTA	CTA	CTA	CTA	CTA	CTA	CTA	CTA	CTA	CTA
K	AAA	AAA	AAA	AAA	AAG	AAG	AAG	AAG	AAG	AAG
I	ATA	ATA	ATA	ATA	ATA	ATA	ATA	ATA	ATA	ATA
H	CAC	CAT	CAC	CAC	NA	CAT	CAC	NA	CAC	NA
G	GGT	GGG	GGA	GGA	GGT	GGA	GGA	GGA	GGA	GGG
F	TTT	TTT	TTT	TTT	TTT	TTC	TTC	TTT	TTC	TTT
E	GAG	GAA	GAA	GAA	GAA	GAG	GAG	GAG	GAG	GAG
D	GAC	GAT	GAT	GAT	GAT	GAC	GAC	GAC	GAC	GAT
C	NA	TGT	NA	NA	TGC	TGC	TGT	TGC	TGT	NA
A	GCT	GCA	GCT	GCA	GCT	GCT	GCA	GCT	GCT	GCA

Position

Cambray

Amino Acid	2	3	4	5	6	7	8	9	10	11
Y	TAT	TAT	TAT	TAC	TAC	TAT	TAC	TAC	TAC	TAC
V	GTT	GTT	GTT	GTT	GTT	GTT	GTT	GTT	GTA	GTT
T	ACT	ACA	ACA	ACA	ACA	ACA	ACA	ACT	ACT	ACT
S	AGT	AGT	TCA	TCA	AGT	AGT	AGC	TCA	AGC	TCG
R	AGA	AGA	CGA	AGA	AGA	AGG	AGA	AGA	AGA	CGG
Q	CAA	CAA	CAA	CAA	CAA	CAG	CAA	CAA	CAA	CAG
P	CCA	CCA	CCA	CCA	CCG	CCC	CCA	CCA	CCC	CCG
N	AAT	AAT	AAT	AAT	AAC	AAT	AAT	AAC	AAT	AAT
L	TTA	TTA	TTA	TTA	CTC	TTA	CTA	TTA	TTG	CTG
K	AAA	AAA	AAA	AAA	AAA	AAA	AAA	AAA	AAA	AAA
I	ATC	ATT	ATA	ATA	ATT	ATA	ATA	ATA	ATT	ATC
H	CAT	CAC	CAC	CAC	CAC	CAC	CAT	CAC	CAC	CAC
G	GGG	GGC	GGT	GGT	GGC	GGA	GGA	GGA	GGT	GGA
F	TTT	TTC	TTT	TTC	TTC	TTT	TTT	TTT	TTT	TTC
E	GAG	GAA	GAA	GAA	GAG	GAA	GAA	GAA	GAA	GAA
D	GAT	GAT	GAT	GAC	GAC	GAT	GAT	GAC	GAC	GAC
C	TGT	TGT	TGT	TGC	TGC	TGT	TGC	TGC	TGC	TGT
A	GCT	GCG	GCT	GCC	GCT	GCC	GCT	GCG	GCT	GCT

Position

Fig S3. Cambray and Goodman most enriched codon, for each amino acid, in highly v lowly expressed transgenes broken down by 5' position. As Fig S2 but using Goodman's "Prot.FCC" metric, rather than "Trans". Position refers to codon position after the start codon.

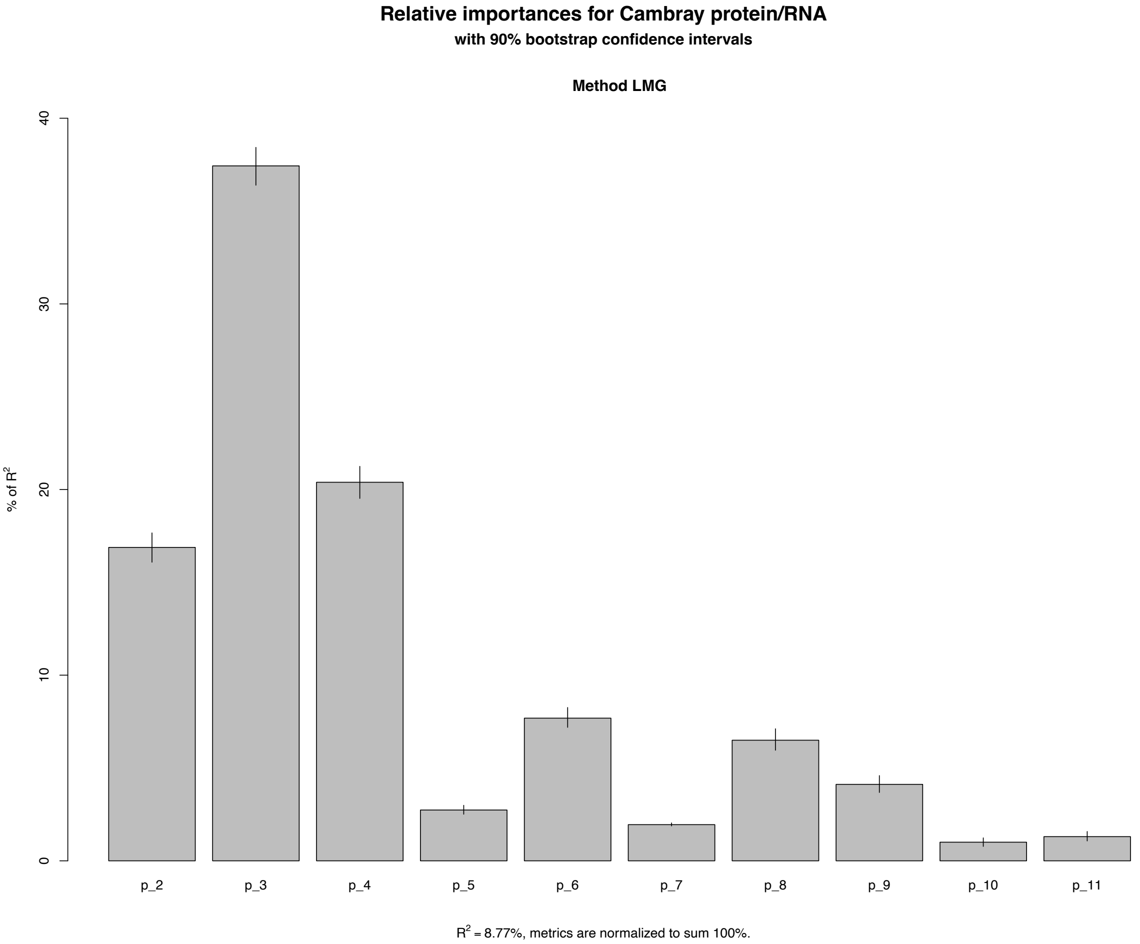

Fig S4. Relaimpo analysis displaying relative influence of each 5' codon position as regards protein/RNA level for Cambray transgene data. Position on the x axis refers to codon position after the start codon. Analysis follows the linear model: Protein per RNA ~ GC codon 2 + GC codon 3 … For information on the relaimpo approach, see Methods and (1).

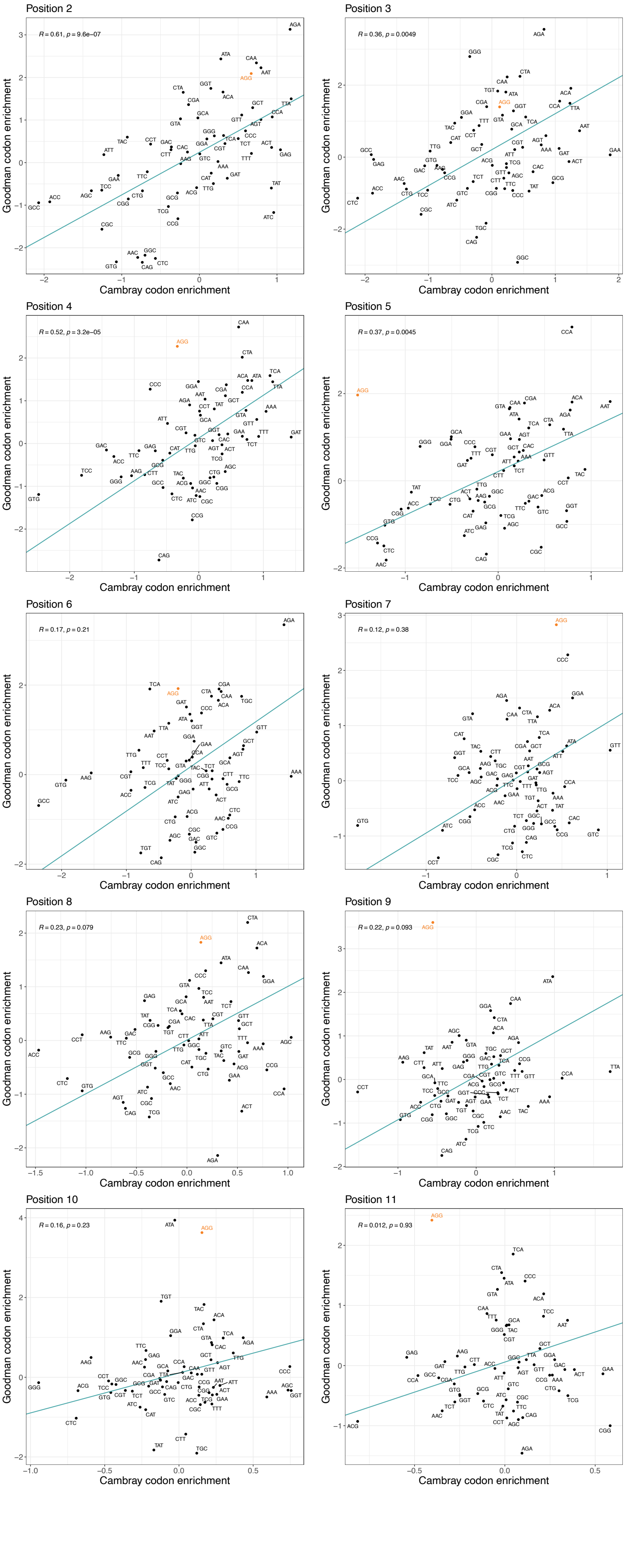
Position 2
R = 0.61, p = 9.6e−07
Position 3
R = 0.36, p = 0.0049
Position 4
R = 0.52, p = 3.2e−05
Position 5
R = 0.37, p = 0.0045
Position 6
R = 0.17, p = 0.21
Position 7
R = 0.12, p = 0.38
Position 8
R = 0.23, p = 0.079
Position 9
R = 0.22, p = 0.093
Position 10
R = 0.16, p = 0.23
Position 11
R = 0.012, p = 0.93
Goodman codon enrichment
Cambray codon enrichment

Fig S5. Cambray v Goodman 5' codon preferences in highly v lowly expressed transgenes broken down by position. Each plot is as in Fig S1C, but in this instance broken down by amino acid position after the start codon. As Fig 3 but using Goodman's "Prot.FCC" metric, rather than "Trans". Regression lines represent a linear model. AGG is marked in orange.

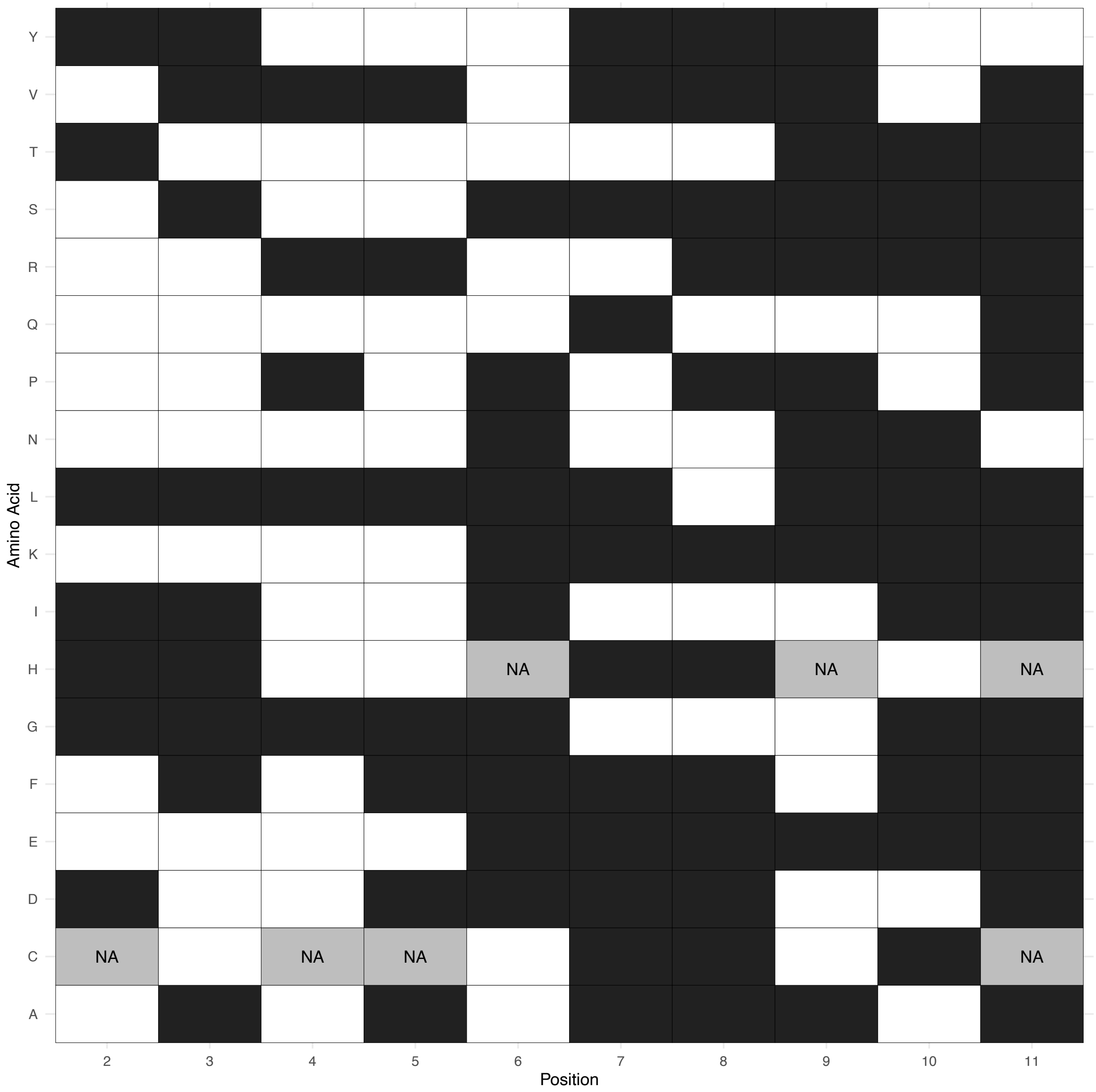

Fig S6. Agreement between Cambray and Goodman 5' codon preferences in highly v lowly expressed transgenes broken down by position, by amino acid. As Fig 4 but using Goodman's "Prot.FCC" metric, rather than "Trans". For Cambray data, the metric for transgene expression is considered as protein per RNA. For a given amino acid (see all listed vertically on the left), the position where the codon with the highest log odds ratio is the same in the two datasets is illustrated in white. Those in disagreement in black. NA is called when any given amino acid is unrepresented in either construct at the given position. For the codons nominated by the two analyses see Fig S3.

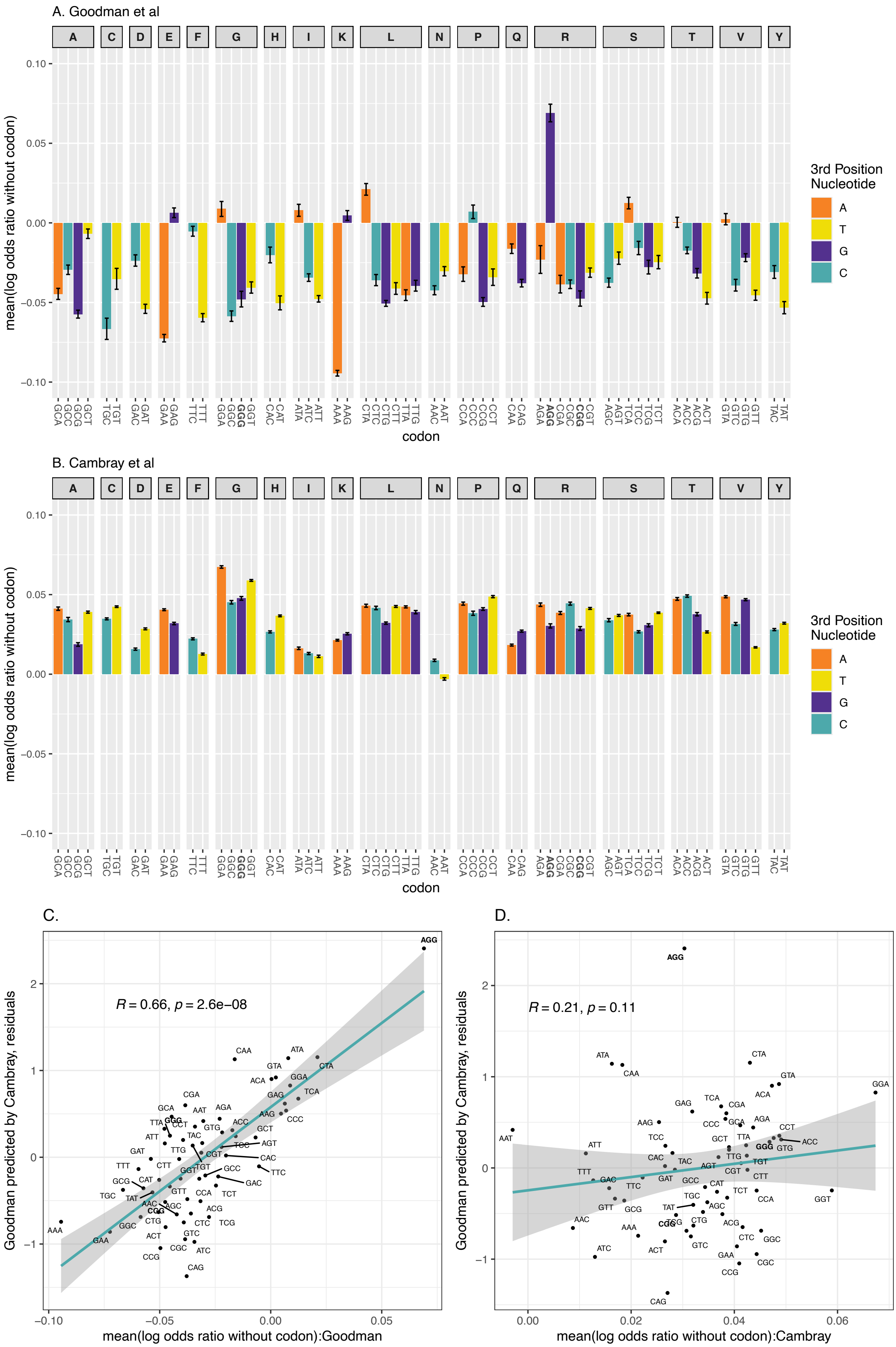
A. Goodman et al
A C D E F G H I K L N P Q R S T V Y
mean(log odds ratio without codon)
codon
3rd Position Nucleotide
A
T
G
C
B. Cambray et al
C.
R = 0.66, p = 2.6e−08
Goodman predicted by Cambray, residuals
mean(log odds ratio without codon):Goodman
D.
R = 0.21, p = 0.11
mean(log odds ratio without codon):Cambray

Fig S7. The relationship between non-random codon allocation to constructs and deviation between datasets using protein/RNA log odds ratios from Cambray data. For the Goodman, using Prot.FCC (**A**) and Cambray, using protein per RNA (**B**) datasets we considered for each construct containing a given codon, the mean log odds ratios of the other codons in the same construct. We calculate the mean of these values for each construct, and then the mean of means for all constructs containing the focal codon. Here we use the log odds ratios derived from the Cambray dataset. The null is a mean of zero. As can be seen, the Goodman data has some highly deviant data points, notably AGG whose flanking codons have a highly positive log odds ratio. To consider whether these deviations explain the differences between Cambray and Goodman log odds ratio we consider the regression between the two. We then determine whether the residuals from this regression are predicted by the mean log odds of the flanking codons in the Goodman data (**C**) and the Cambray data (**D**). Statistics in C and D are Spearman rank correlations. Error bars in panels A and B are standard error (SEM). NGG codons are highlighted in bold.

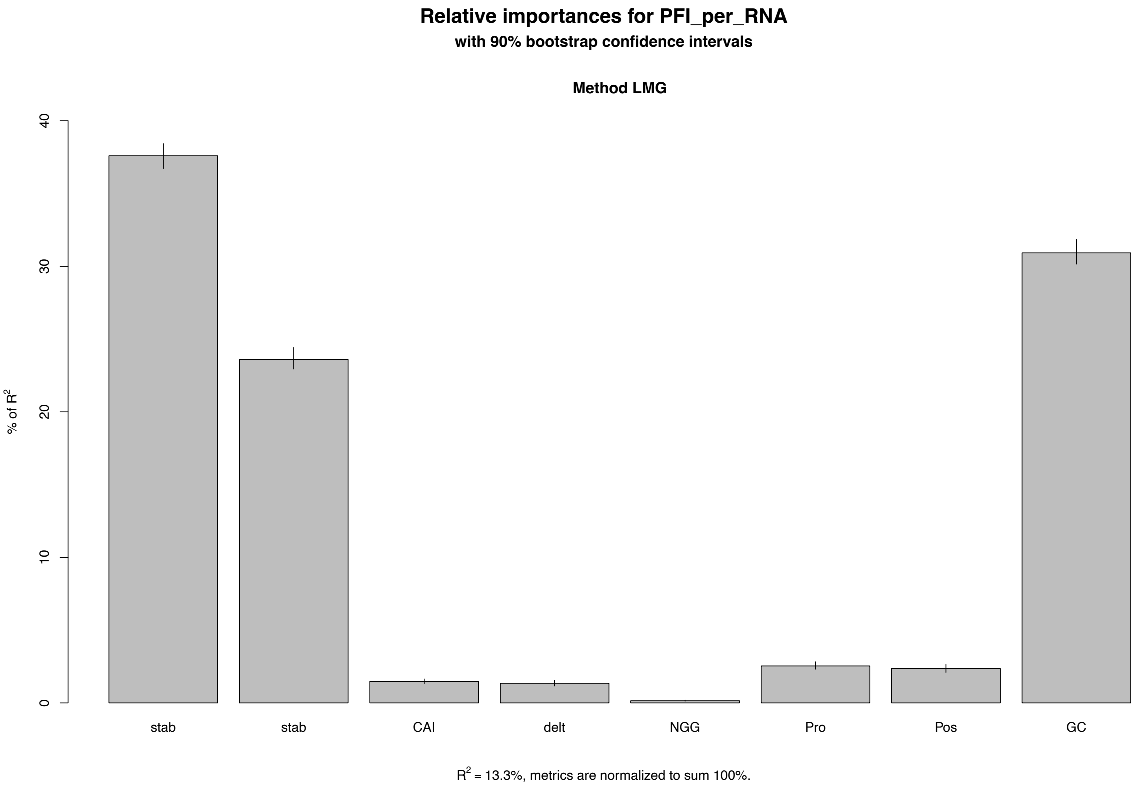

Fig S8. Relaimpo analysis displaying relative influence of potential predictors as regards protein/RNA level for Cambray transgene data. Protein per RNA refers to production with facilitated translation initiation (PFI/RNA_{SS} in Cambray data). The first stab measure on the x axis is the stability of the construct from -30 to +30, whilst the second stab measure is stability for 0 to +60bp, CAI is the log odds ratio for each codon reflecting enrichment in the core of highly expressed native genes, assayed using protein abundance, delta CAI is the difference between the CAI metric for 5' ends (taken to be the first 10 codons) and that of the rest of the gene, NGG is the density of NGG codons in the first 6 codons (where N is any of the four nucleotides), Pro is proline density in the first 10 codons, Pos the density of positively charged amino acids, and GC is the G+C nucleotide content of the first 10 codons. Analysis follows the linear model: PFI per RNA ~ stab30 + stab60 + CAI + delta CAI + NGG + Pro +Pos + GC. For information on the relaimpo approach, see Methods and (1).

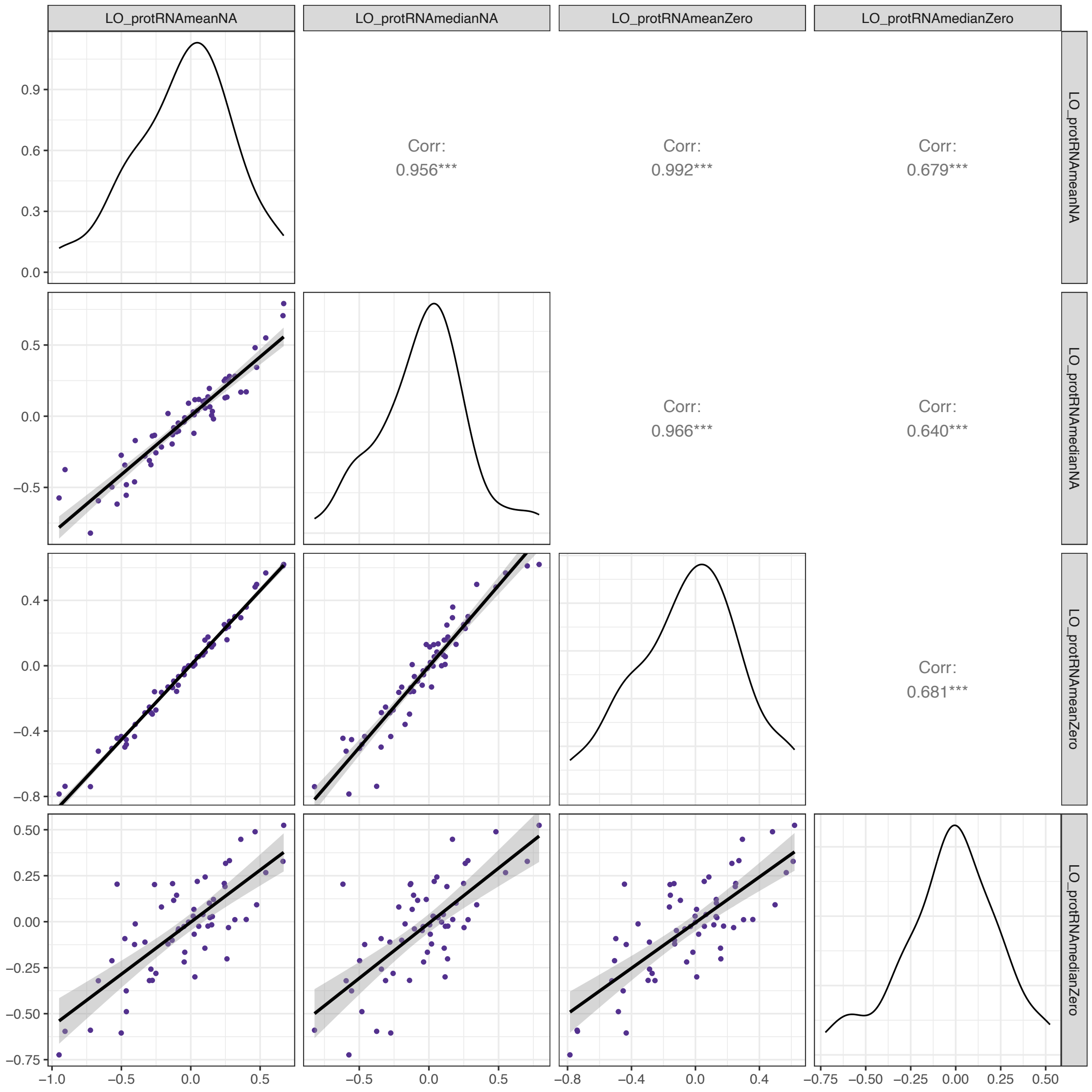

Fig S9. Correlation matrix with pairwise comparison of log odds ratios for native protein/RNA enrichment. We considered native RNA data in 4 different ways: mean of all measures with no data treated as zero (LO_protRNAmeanZero); mean of all measures with no data treated as NA (LO_protRNAmeanNA), median of all measures with no data treated as zero (LO_protRNAmedianZero); median of all measures with no data treated as NA (LO_protRNAmedianNA). Bottom quadrant represents a linear regression, upper quadrant is the Spearman correlation with stars to signal significance, along the diagonal is data distribution. All native protein data was obtained from PaxDB (2) and cross-referenced with RNA measures from a compendium of RNA-seq datasets (3).

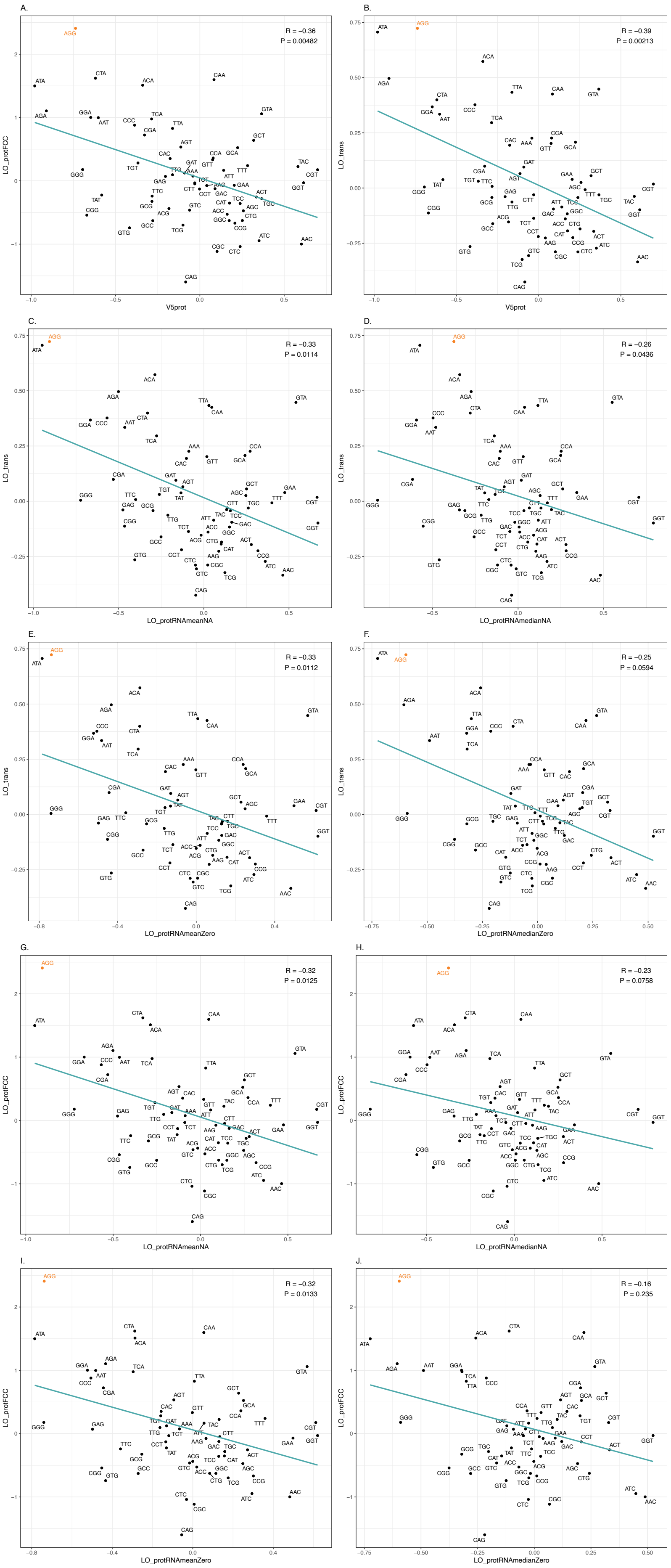
A.
R = −0.36
P = 0.00482
LO_protFCC
V5prot
B.
R = −0.39
P = 0.00213
LO_trans
V5prot
C.
R = −0.33
P = 0.0114
LO_trans
LO_protRNAmeanNA
D.
R = −0.26
P = 0.0436
LO_trans
LO_protRNAmedianNA
E.
R = −0.33
P = 0.0112
LO_trans
LO_protRNAmeanZero
F.
R = −0.25
P = 0.0594
LO_trans
LO_protRNAmedianZero
G.
R = −0.32
P = 0.0125
LO_protFCC
LO_protRNAmeanNA
H.
R = −0.23
P = 0.0758
LO_protFCC
LO_protRNAmedianNA
I.
R = −0.32
P = 0.0133
LO_protFCC
LO_protRNAmeanZero
J.
R = −0.16
P = 0.235
LO_protFCC
LO_protRNAmedianZero

Fig S10. Comparison of codon enrichment log odds ratios between Goodman and native genes. We firstly (**A**) replicate the prior result by Lewin et al. (4) that employed native protein level and Goodman's Prot.FCC (LO_protFCC), and (**B**) repeat it for native protein level and Goodman's Trans (LO_trans). For both Goodman's Trans (**C-F**) and Prot.FCC (**G-J**), we then examine native protein/RNA measures considering native RNA data in 4 ways: mean of all measures with no data treated as zero (LO_protRNAmeanZero); mean of all measures with no data treated as NA (LO_protRNAmeanNA), median of all measures with no data treated as zero (LO_protRNAmedianZero); median of all measures with no data treated as NA (LO_protRNAmedianNA). Plots display a linear regression and Spearman correlation with respective p-value. All native protein data was obtained from PaxDB (2) and cross-referenced with RNA measures from a compendium of RNA-seq datasets (3).

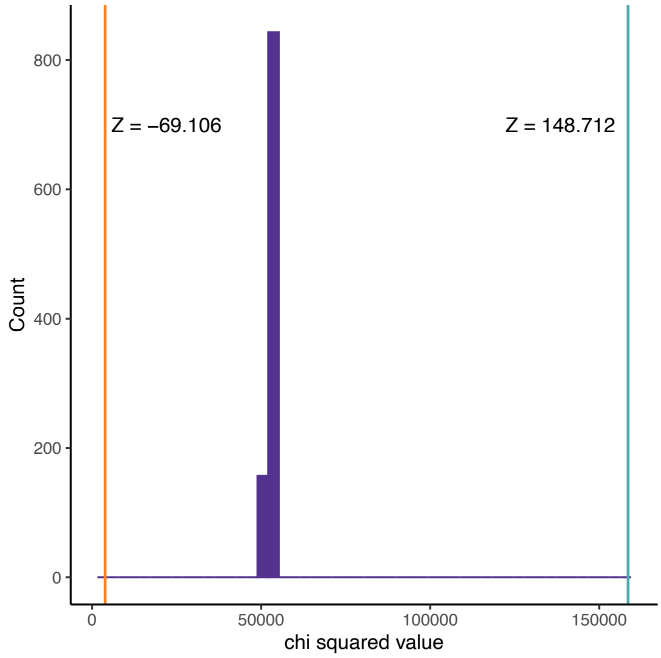

Fig S11. Evidence of passenger effects affecting Goodman and Cambray datasets, even when considering Cambray constructs with unique 5' CDS. Chi squared values of observed co-occurrences between a focal codon and any given non focal codon under a null of random association. For each codon we consider those constructs containing it and determine the count of each non-focal codon in the same constructs. We determine the expected value by considering the total number of non-focal codons in the same constructs and multiplying by the frequency (amongst the non-focal codons) in the dataset as a whole of each non-focal codon, i.e. an assumption of random association. As chi squared is sensitive to total counts, we randomly subsample 14,234 sequences from the Cambray set for 1000 times, the distribution of which is the histogram. The orange line indicates the 1% significance level with df = 3659. The light blue line is the sum chi squared value for the Goodman data.

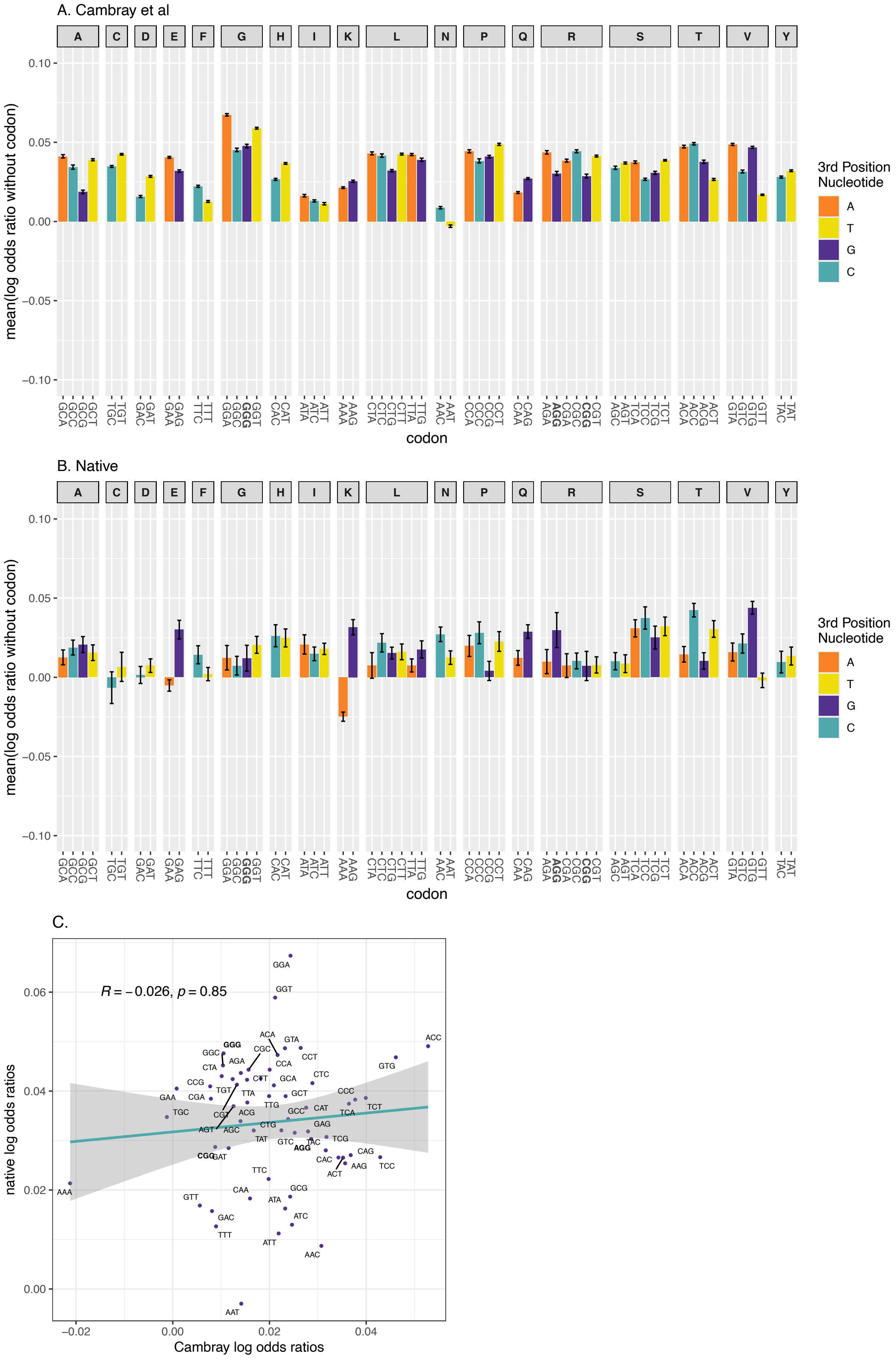
A. Cambray et al
A C D E F G H I K L N P Q R S T V Y
mean(log odds ratio without codon)
0.10
0.05
0.00
−0.05
−0.10
codon
3rd Position Nucleotide
A
T
G
C
B. Native
A C D E F G H I K L N P Q R S T V Y
mean(log odds ratio without codon)
0.10
0.05
0.00
−0.05
−0.10
codon
3rd Position Nucleotide
A
T
G
C
C.
R = −0.026, p = 0.85
native log odds ratios
0.06
0.04
0.02
0.00
−0.02
0.00
0.02
0.04
Cambray log odds ratios

Fig S12. The relationship between non-random codon allocation to constructs and deviation in Cambray and native data using protein/RNA log odds ratios from Cambray data. For the Cambray (**A**) and native (**B**) datasets we considered for each construct containing a given codon, the mean log odds ratios of the other codons in the same construct. We calculate the mean of these values for each construct and then the mean of means for all constructs containing the focal codon. Here we use the log odds ratios derived from the Cambray dataset. The null is a mean of zero. Error bars in both panels A and B represent standard error (SEM). **C.** Comparison of Cambray (protein/RNA) and native (protein) codon enrichment vectors. Native protein data was obtained from PaxDB (2). NGG codons are highlighted in bold.

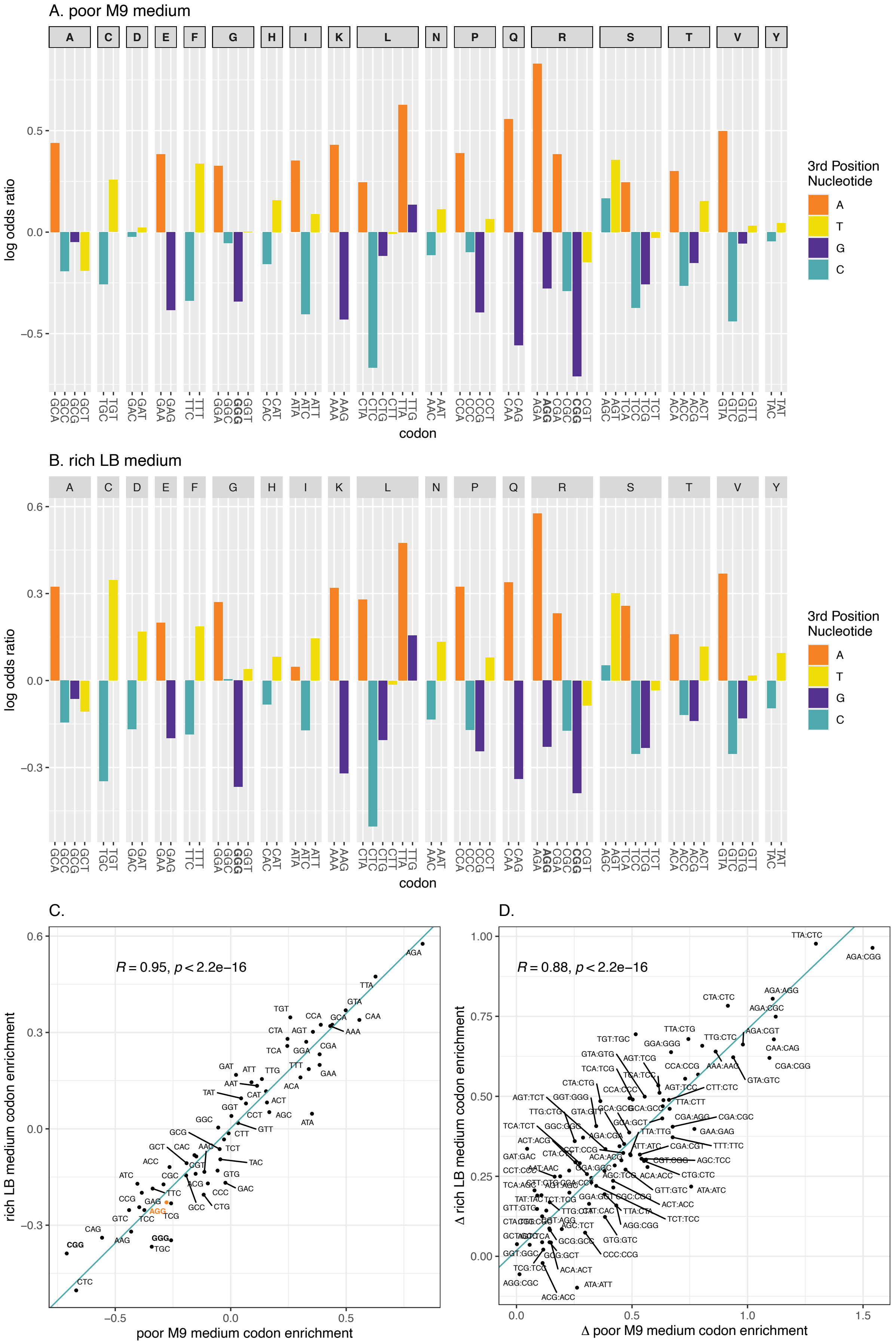

A. poor M9 medium
log odds ratio
codon
3rd Position Nucleotide
A
T
G
C
B. rich LB medium
log odds ratio
codon
3rd Position Nucleotide
A
T
G
C
C.
R = 0.95, p < 2.2e−16
rich LB medium codon enrichment
poor M9 medium codon enrichment
D.
R = 0.88, p < 2.2e−16
Δ rich LB medium codon enrichment
Δ poor M9 medium codon enrichment

Fig S13. Osterman rich v poor media 5' codon preferences in highly v lowly expressed transgenes. Log odds ratios for enrichment in the top quartile by translation efficiency (TEF in Osterman et al (5), see Methods) for each codon within a synonymous codon block for cells grown in **A.** poor M9 medium data and **B.** rich LB medium. **C.** The x axis is the log odds ratio for the codon being enriched at the 5' ends in poor M9 medium transgenes with high TEF compared to low TEF, the y axis is for rich LB medium. Each data point is labelled as the codon it represents, AGG is marked in orange. For panels A, B and C, NGG codons are highlighted in bold. **D.** As for panel C, but comparing all pairwise combinations of synonymous codons (i.e. within the same codon block: N=87). The pairwise differences are oriented such that, on the x axis, the codon with the lower value of the log odds ratio has its value subtracted from that of the higher value. The orientation is preserved for the y axis. Each point is labelled by the oriented codon pair (first codon in the pair has the higher x axis value, as seen in panel C). For both C and D, Principal Components Analysis (PCA) was used to fit an orthogonal regression line, and the Pearson correlation coefficient and p-value are provided within the plots.

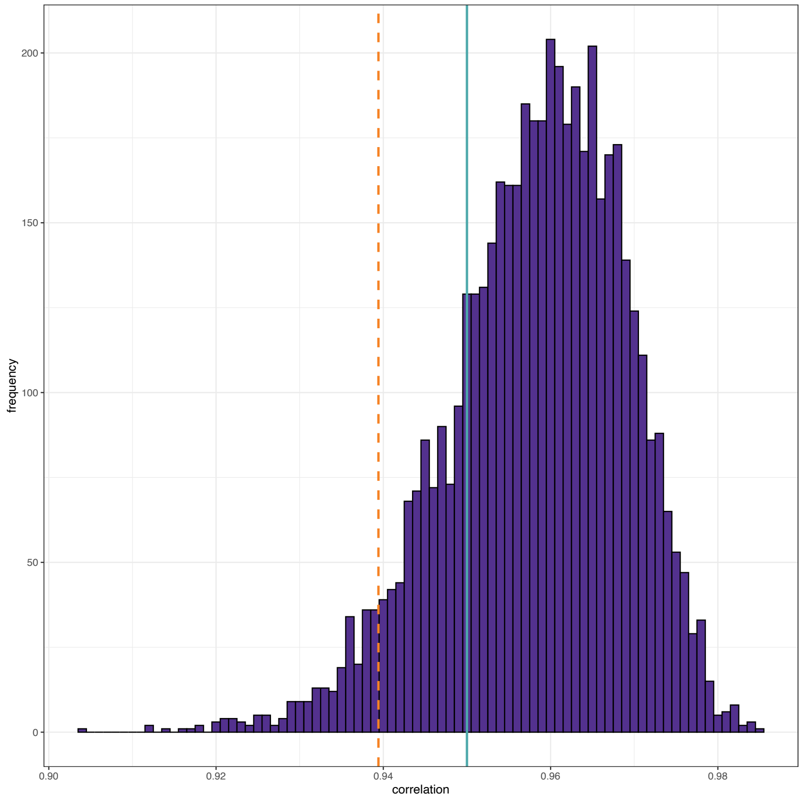

Fig S14. Histogram of Pearson correlations between 100 bootstrapped calculations of log odds ratios for codon enrichment in highly v lowly expressed transgenes. Codon enrichment was found considering Osterman et al (5) constructs expressed in cells grown in rich LB medium, with high and low expression determined based on their Translation Efficiency Fractions (TEF) as described in Methods. Log odds ratio calculations were repeated 100 times using bootstrapping with replacement when sampling constructs from each TEF group. This approach yields a distribution of log odds ratios for each codon rather than a single estimate. Each possible pairwise comparison among bootstraps was used to compute Pearson correlations. The vertical solid light blue line marks a Pearson correlation of 0.95 (the correlation between codon enrichment patterns in rich LB and poor M9 media, see Fig S13C). The vertical dashed orange line indicates the significance threshold at $P < 0.05$ (the 95% bootstrap bound).

Supplementary Tables
(provided as separate files)

Table S1. Codon enrichment log odds ratios for a range of metrics across the data of Lewin et al, Cambray et al and Goodman et al. From Lewin et al (4): V5prot is the 5' codon enrichment in the native gene data (top 25% by protein abundance v bottom 25%) as indicated by PaxDB (2); VedIO is the 5' enrichment in the experimental transgene data reported by Goodman et al (top 25% by "Prot.FCC" v bottom 25%). From Cambray et al (6): LO_PNInorm is the 5' enrichment in the experimental transgene protein/RNA data using protein measures under normal translation initiation (top 25% by "PNI"/"RNAss" v bottom 25%); is the 5' enrichment in the experimental transgene protein/RNA data using protein measures under facilitated translation initiation (top 25% by "PFI"/"RNAss" v bottom 25%); LO_utrCdsStructureMFE is the 5' enrichment in the reported Minimum Free Energy level of the mRNA structure from position -30 to +30 within constructs with constant sequences from -30 to +3 (top 25% by "gs.utrCdsStructureMFE" v bottom 25%); LO_fivepCdsStructureMFE is 5' enrichment in the reported Minimum Free Energy level of the mRNA structure from position 0 to +60 (top 25% by "gs.fivepCdsStructureMFE" v bottom 25%); LO_RNAfold_C is 5' enrichment in the RNA stability measure generated by the ViennaRNA R package (7) for the segment of 10 codons following the start codon of Cambray et al sequences. From Goodman et al (8): LO_protFCC is 5' enrichment in the experimental transgene protein data (top 25% by "Prot.FCC" v bottom 25%, which is equal to VedIO from Lewin et al above); LO_trans is 5' enrichment in the experimental transgene protein/RNA data (top 25% by "Trans" v bottom 25%); LO_dG is 5' enrichment in RNA folding energy of the entire transcript, from the TSS to +96 (top 25% by "dG" v bottom 25%); LO_dGnoutr is 5' enrichment in RNA folding energy of the transcript starting at the beginning of the CDS (top 25% by "dG.noutr" v bottom 25%); LO_dGunif is 5' enrichment in RNA folding energy of the transcript cut off at position -5 (top 25% by "dG.unif" v bottom 25%); LO_RNAfold_G is 5' enrichment in the RNA stability measure generated by the ViennaRNA R package (7) for the segment of 10 codons following the start codon of Goodman et al sequences. For native genes, the 5' enrichment was calculated for top v bottom 25% of protein by RNA (protein abundance was retrieved from PaxDB (2) and RNA measures from a compendium of RNA seq datasets (3)), considering RNA measures in 4 different ways: mean of all measures with no data treated as zero (LO_protRNAmeanZero); mean of all measures with no data treated as NA (LO_protRNAmeanNA), median of all measures with no data treated as zero (LO_protRNAmedianNA); median of all measures with no data treated as NA (LO_protRNAmedianNA). From Osterman et al (5): LO_richLB is 5' enrichment in the experimental translation efficiency data (top 25% by "TEF" v bottom 25%) for cells grown in rich LB medium; LO_poorM9 is 5' enrichment in the experimental translation efficiency data (top 25% by "TEF" v bottom 25%) for cells grown in poor M9 medium.

Table S2. Spearman correlation matrix comparing the log odds calculated from all stability and protein measures between Cambray and Goodman data. Significance is displayed with a ranging number of stars so that: p < .0001 = "****"", p < .001 = "*** ", p < .01 = "** ", p < .05 = "* ", p > .05 = no stars. From Cambray et al (6): LO_PNInorm is the 5' enrichment in the experimental transgene protein/RNA data (top 25% by "PNI"/"RNAss" v bottom 25%); LO_utrCdsStructureMFE is the 5' enrichment in the reported Minimum Free Energy level of the mRNA structure from position -30 to +30 within constructs with constant sequences from -30 to +3 (top 25% by "gs.utrCdsStructureMFE" v bottom 25%); LO_fivepCdsStructureMFE is 5' enrichment in the reported Minimum Free Energy level of the mRNA structure from position 0 to +60 (top 25% by "gs.fivepCdsStructureMFE" v bottom 25%); LO_RNAfold_C is 5' enrichment in the RNA stability measure generated by the ViennaRNA R package (7) for the segment of 10 codons following the start codon of Cambray et al sequences. From Goodman et al (8): LO_protFCC is 5' enrichment in the experimental transgene protein data (top 25% by "Prot.FCC" v bottom 25%); LO_trans is 5' enrichment in the experimental transgene protein/RNA data (top 25% by "Trans" v bottom 25%); LO_dG is 5' enrichment in RNA folding energy of the entire transcript, from the TSS to +96 (top 25% by "dG" v bottom 25%); LO_dGnoutr is 5' enrichment in RNA folding energy of the transcript starting at the beginning of the CDS (top 25% by "dG.noutr" v bottom 25%); LO_dGunif is 5' enrichment in RNA folding energy of the transcript cut off at position -5 (top 25% by "dG.unif" v bottom 25%); LO_RNAfold_G is 5' enrichment in the RNA stability measure generated by the ViennaRNA R package (7) for the segment of 10 codons following the start codon of Goodman et al sequences.

Table S3. Pearson correlations and the respective significance between the log odds ratios of 5' v core codon enrichment of 1355 bacterial genomes and the codon enrichment in highly v lowly expressed transgenes in the Goodman and Cambray data. "assembly_accession" refers to the accession number for sequence retrieval from RefSeq NCBI (9).

Table S4. Correlations and partial Spearman's correlations of potential predictors on cell growth rates in Cambray data. Fitness refers to growth rate with normal translation initiation (WNI from Cambray data). Stab30 is the stability of the construct from -30 to +30, likewise stab60 for 0 to +60bp (both metrics reported by Cambray et al), CAI is the log odds ratio for each codon reflecting enrichment in the core of highly expressed native genes, assayed using protein abundance (see (4)), NGG is the density of NGG codons in the first 6 codons (where N is any of the four nucleotides), Pro is proline density in the first 10 codons, Pos the density of positively charged amino acids, and GC is the G+C nucleotide content of the first 10 codons. Significance is displayed with a ranging number of stars so that: p < .0001 = "****"", p < .001 = "*** ", p < .01 = "** ", p < .05 = "* ", p > .05 = no stars. The upper quadrant along the table diagonal is for correlations, the lower for the partial correlations. The first column is the prediction of protein per RNA allowing for covariation.

Table S5. Correlations and partial Spearman's correlations of potential predictors on protein per RNA in Cambray data. Protein per RNA refers to production with facilitated translation initiation (PFI/RNA_{SS} from Cambray data). Stab30 is the stability of the construct from -30 to +30, likewise stab60 for 0 to +60bp (both metrics reported by Cambray et al), CAI is the log odds ratio for each codon reflecting enrichment in the core of highly expressed native genes, assayed using protein abundance (see (4)), NGG is the density of NGG codons in the first 6 codons (where N is any of the four nucleotides), Pro is proline density in the first 10 codons, Pos the density of positively charged amino acids, and GC is the G+C nucleotide content of the first 10 codons. Significance is displayed with a ranging number of stars so that: p < .0001 = "****"", p < .001 = "*** ", p < .01 = "** ", p < .05 = "* ", p > .05 = no stars. The upper quadrant along the table diagonal is for correlations, the lower for the partial correlations. The first column is the prediction of protein per RNA allowing for covariation.

References (for supplementary material)

1. Groemping, U. (2006) Relative importance for linear regression in R: the package relaimpo. *Journal of Statistical Software*, **17**.
2. Huang, Q., Szklarczyk, D., Wang, M., Simonovic, M. and von Mering, C. (2023) PaxDb 5.0: curated protein quantification data suggests adaptive proteome changes in yeasts. *Molecular & Cellular Proteomics*, **22**.
3. Tjaden, B. (2023) Escherichia coli transcriptome assembly from a compendium of RNA-seq data sets. *Rna Biology*, **20**, 77-84.
4. Lewin, L.E., Daniels, K.G. and Hurst, L.D. (2023) Genes for highly abundant proteins in Escherichia coli avoid 5' codons that promote ribosomal initiation. *Plos Computational Biology*, **19**.
5. Osterman, I.A., Chervontseva, Z.S., Evfratov, S.A., Sorokina, A.V., Rodin, V.A., Rubtsova, M.P., Komarova, E.S., Zatsepin, T.S., Kabilov, M.R., Bogdanov, A.A. *et al.* (2020) Translation at first sight: the influence of leading codons. *Nucleic Acids Research*, **48**, 6931-6942.
6. Cambray, G., Guimaraes, J.C. and Arkin, A.P. (2018) Evaluation of 244,000 synthetic sequences reveals design principles to optimize translation in Escherichia coli. *Nature Biotechnology*, **36**, 1005-+.
7. Lorenz, R., Bernhart, S.H., Siederdissen, C.H.Z., Tafer, H., Flamm, C., Stadler, P.F. and Hofacker, I.L. (2011) ViennaRNA package 2.0. *Algorithms for Molecular Biology*, **6**.
8. Goodman, D.B., Church, G.M. and Kosuri, S. (2013) Causes and effects of N-terminal codon bias in bacterial genes. *Science*, **342**, 475-479.
9. O'Leary, N.A., Wright, M.W., Brister, J.R., Ciufo, S., McVeigh, D.H.R., Rajput, B., Robbertse, B., Smith-White, B., Ako-Adjei, D., Astashyn, A. *et al.* (2016) Reference sequence (RefSeq) database at NCBI: current status, taxonomic expansion, and functional annotation. *Nucleic Acids Research*, **44**, D733-D745.
